# Supplementary material for: Business-as-usual and fantasy planning – an analysis of equity within climate adaptation planning for sanitation in Nairobi
Source: PLoS One. 2025 Dec 30;20(12):e0339272. doi: 10.1371/journal.pone.0339272 (PMC12752985; doi:10.1371/journal.pone.0339272)
Supplement: S1 Table — (PDF) [file pone.0339272.s001.pdf]

SUPPLEMENTAL INFORMATION: Business-as-usual and fantasy planning – an analysis of equity withing climate adaptation planning in Nairobi

S1 Table: Summary of reviewed climate change policy and planning framework documents with focus on specific references to sanitation system adaptation and equity considerations

Climate Change Policy and Planning Framework	Sanitation adaptation specific commitments	Main findings and remarks on equity considerations
National		
National Climate Change Response Strategy (NCCRS, 2010) (1)	Only vague references to sanitation	*Recognition:* Acknowledges that climate change will largely affect population of poor urban neighbourhoods
National Climate Change Action Plan (NCCAP 2018 - 2022) (2)	Focuses on vector control, flood resilience, and solid waste management under 'Health, Sanitation and Human Settlements'.	'Equity and social inclusion' is one of the four guiding principles of the NCCAP but there are no specific considerations of equity in the sanitation relevant sections
National Adaptation Plan (NAP) 2015 -2030 (3)	Vague actions for water and sanitation, linking to the National Water Master Plan for long-term actions.	*Procedures:* Under the Water and Sanitation sector section, the NAP acknowledges that the sector needs to identify current and future vulnerabilities
National Determined Commitments (NDC) 2020 update (4)	No specific plans for sanitation sector adaptation; mentions future climate risk assessments for water, sanitation and irrigation infrastructure	*Procedures:* The NDC makes the claim that in terms of 'just transitions' Kenya follows an extensive consultation process to ensure involvement by all stakeholders
National Policy on Climate Finance 2016 (5)	No priority actions for sanitation; mentions potential use of climate finance for sewerage infrastructure under disaster risk management section.	*Distribution:* The policy goal is to ensure equitable benefit sharing from climate change interventions in the country but there are no specific equity considerations under the sections relevant for sanitation
The Climate Change Act (2016) (6) and The Climate Change (Amendment) Bill (2023) (7)	No specific reference to water and sanitation sector	Only unspecific references to need to ensure social and intergenerational equity
Nairobi City County		
Nairobi City County Climate Action Plan 2020 – 2050 (8)	Claims strong capacity in enhancing water and wastewater/sanitation resilience, but lacks clarity on implementation. Plans for new wastewater management systems and methane recovery; however, lacks budgeted plans and tangible improvements. Includes development of by-laws to prohibit new open drains and rehabilitation of stormwater drains to reduce urban flooding. Sub-action for strengthening climate-related disease mapping includes increasing sanitation facilities in vulnerable areas.	*Distribution:* Refers to severe pollution of Nairobi rivers which is partly due to illegal discharge of sewage / sludge<br><br>*Recognition:* Recognises risk of flooding for low-income population living in flood prone areas

References

1. GoK. National Climate Change Response Strategy. Nairobi, Kenya: Government of Kenya; 2010.
2. GoK. National Climate Change Action Plan (Kenya) 2018-2022. Nairobi, Kenya: Ministry of Environment and Forestry; 2018.
3. MoENR. Kenya National Adaptation Plan 2015-2030. Nairobi, Kenya: Ministry of Environment and Natural Resources; 2016.
4. MoEF. Nationally Determined Contribution: Kenya. 2020 update. Nairobi, Kenya: Ministry of Environment and Forestry; 2020.
5. GoK. National Policy on Climate Finance. Nairobi, Kenya: The National Treasury; 2016.
6. RoK. The Climate Change Act. Nairobi, Kenya: Republic of Kenya; 2016.
7. GoK. The Climate Change (Amendment) Bill, 2023. In: GoK, editor. Nairobi, Kenya2023.
8. NCC. Climate Action Plan 2020-2050. Nairobi, Kenya: Nairobi City County; 2020.
