# Supplementary material for: Business-as-usual and fantasy planning – an analysis of equity within climate adaptation planning for sanitation in Nairobi
Source: PLoS One. 2025 Dec 30;20(12):e0339272. doi: 10.1371/journal.pone.0339272 (PMC12752985; doi:10.1371/journal.pone.0339272)
Supplement: S2 Table — (PDF) [file pone.0339272.s002.pdf]

SUPPLEMENTAL INFORMATION: Business-as-usual and fantasy planning – an analysis of equity withing climate adaptation planning in Nairobi

S2 Table. *Summary of reviewed Sanitation Sector Policy and Planning Framework Documents with focus on specific references to climate change adaptation and equity considerations*

Sanitation sector Policy and Planning Framework	Adaptation to climate change relevant considerations	Main findings and remarks on equity considerations
National		
Kenyan Water Act (1)	No specific link between climate change adaptation and sanitation.	*Distribution:* Only covers sewerage services; refers to sewerage levy for service extensions
National Water Master Plan 2013 (2)	Focuses on water resource management; lacks specific mention of climate adaptation in relation to sanitation in the Athi Catchment.	*Distribution:* Mainly sewerage; plan mentions onsite sanitation but lacks details for non-sewered sanitation
National Water and Sanitation Service Strategy (2020 -2025) (3)	Recognizes relevance of Climate Change Act; considers climate change a crosscutting issue.	*Distribution:* Aims for universal access to sanitation, including sewerage and non-sewerage; onsite sanitation mainly for peri-urban and rural areas<br>*Recognition:* Refers to sanitation levy to help bridge funding gap for sanitation
Pro-poor Implementation Plan for Water Supply and Sanitation 2007 (4)	No reference to climate change	*Distribution:* Advocates gradually replacing informal with formal services provision<br>*Recognition:* Stipulates pro-poor orientation of sector and prioritisation of services for urban poor
Kenya Environmental Sanitation and Hygiene Policy 2016 -2030 (5)	Lacks concrete guidance on climate change adaptation but includes some limited guidance on flood control and drainage.	*Distribution:* Acknowledges onsite and offsite systems and range of systems for onsite<br>*Recognition:* Acknowledges lack of access to sanitation services for the poor. Recommends that government should support implementation of urban environmental sanitation<br>*Procedures:* Emphasizes community involvement in technology choices.
National Environmental Health and Sanitation Bill (2020)	*Not available*	
Sector Plan for Environment, Water, Sanitation And Regional Development (2018 -2022) (6)	Climate change is recognised as emerging issue but not specifically linked to any of the sanitation actions	*Distribution:* Only unspecific references to low-income area sanitation services provision<br>*Procedures:* States that the preparation of the Medium Term Plan III has followed an participatory planning process including organisations representing vulnerable groups
Prototype County Environmental Health and Sanitation Bill 2016 (7)	No reference to climate change	*Distribution:* Stresses equitable access to sanitation as part of the guiding principles. Emphasizes duty of County Governments to allocate resources for sanitation services equitably<br>*Procedures:* Stresses importance of public participation in planning

**Sanitation sector Policy and Planning Framework**	**Adaptation to climate change relevant considerations**	**Main findings and remarks on equity considerations**
National ODF Kenya 2020 Campaign Framework 2016/2017 – 2019/20 (8)	No reference to climate change	*No specific equity considerations*
National Sanitation Management Policy (currently debated in parliament) (9)	Acknowledges impacts of climate change on sanitation but lacks specific action plans.	*Distribution:* Aims for universal, equitable, sustainable sanitation services across the entire chain'<br>*Procedures:* Describes participatory process of the policy formulation
WASREB Guidelines On Sanitation Levy And Trade Effluent Surcharge By Water Service Providers In Kenya 2019 (10)	No reference to climate change	*Distribution:* Mentions Kenya's adoption of Countywide inclusive sanitation and addresses full sanitation service chain. Stipulates equitable levy burden<br>Recognition: The principles of Countywide Inclusive Sanitation include targeted measures for the poor.
WASREB Guidelines for Provision of Water and Sanitation Services In Rural and Underserved Areas In Kenya 2016 (11)	No reference to climate change	*No specific equity considerations*
WASREB Water Service Performance Report 2021/22 Impact 15 (2022) (12)	Climate change adaptation is not specifically linked to sanitation. WASREB has no performance indicator for climate resilience of service provider	Since 2018 WASREB requires the WSPs to report on a set of Pro-poor indicators however this assessment does not form part of the overall performance ranking
Nairobi City County		
Nairobi City County Policy on Water and Sanitation 2018 (13)	*Only Sessional Paper could be reviewed.*<br>Mentions mainstreaming climate change adaptation and mitigation in water and sanitation management.	*Distribution:* Focuses on sewerage; however states that NCC should subsidises water and sanitation services in low-income areas
Nairobi City County Water and Sanitation Act (2019)	*Not available*	
Nairobi Water and Sewerage Company Strategic Plan 2019/20 - 2023/24 (14)	Climate change mentioned only as a risk factor for reduced raw water supply, no direct link to sanitation.	*Distribution:* Refers to surge of residential and commercial development that has put additional strain on water and sanitation infrastructure
Strategic Guidelines for Improving Water and Sanitation Services in Nairobi's Informal Settlements 2009 (15)	No reference to climate change	*Distribution:* Suggests that NCWSC will promote on-site sanitation in areas that are outside sewered area; however this is not in line with current mandate of NCWSC<br>*Procedures:* Suggests to s systematize and increase the transparency of WSS interventions for recipients and partners in informal settlements.
Athi Water Works Development Agency – Strategic Plan: 2018 -2022 (16)	Climate change is not explicitly linked to sanitation (references focus on water resources)	Distribution: AWWDA is only responsible for sewerage and does not engage in promotion or implementation of non-sewered sanitation *No specific equity considerations*
**Other relevant documents**		
National		

Sanitation sector Policy and Planning Framework	Adaptation to climate change relevant considerations	Main findings and remarks on equity considerations
Constitution of Kenya (17)	Article 43(1) b and 43(1) d provides that access to reasonable standards of sanitation and clean safe water in adequate quantities is an economic and social right to every person.	
Kenya Vision 2030 Third Medium Term Plan (MTP III, 2018 – 2022) (18)	There are no concrete programmes linking sanitation and climate change for the period 2018 -2022.	*Distribution:* Vision 2030 commits to universal water and sanitation access by the year 2030'.<br>*Procedures:* States that MTP III has been prepared through inclusive planning process including organisations representing vulnerable groups
Nairobi County		
Nairobi City County Integrated Development Plan (CIDP) 2023-2027 (19)	There are no concrete links made between climate adaptation and sanitation and there are no climate resilience, adaptation or mitigation strategies for the sanitation sector	*Distribution:* Refers to severe pollution of Nairobi rivers which is partly due to illegal discharge of sewage / sludge<br>*Recognition:* Acknowledges sanitation service gaps in slums<br>*Procedures:* Acknowledges low level of public inclusivity of last CIDP/ NCC activities in general but there are not concrete actions for improvement during implementation of current CIDP
Nairobi County Annual Development Plan 2022/23 (20)	No linkage between climate adaptation and sanitation	*Distribution:* Mentions NCWSC commitment to constitutional right to water and sanitation and commitment to improving water and sewer networks and connectivity but does not explicitly explain sanitation improvements beyond sewer networks<br>Refers to severe pollution of Nairobi rivers which is partly du e to illegal discharge of sewage / sludge
Nairobi Integrated Urban Development Masterplan (NIUPLAN) (21)	No specific actions for climate change adaptation under the stormwater drainage and sewerage section	*Distribution:* Refers to severe pollution of Nairobi rivers which is partly due to illegal discharge of sewage / sludge<br>*Recognition:* Acknowledges environmental, legal and economic vulnerability of people living in urban low income areas

References:

1. GoK. Water Act. In: Kenya GotRo, editor. Nairobi: Government of the Republic of Kenya; 2016.
2. MoEWNR and WRMA. The National Water Master Plan 2030. In: Ministry of Environment WaNRWRMA, editor. Nairobi, Kenya: Republic of Kenya; 2013.
3. MoWSI. National Water and Sanitation Strategy 2020-2025. In: Ministry of Water SaI, editor. Nairobi, Kenya: Republic of Kenya; 2021.
4. MoWI. The Pro-Poor Implementation Plan for Water Supply and Sanitation (PPIP - WSS). Nairobi, Kenya: Ministry of Water and Irrigation; 2007.
5. MoH. Kenya Environmental Sanitation and Hygiene Policy 2016 – 2030. Nairobi, Kenya: Ministry of Health; 2016.
6. GoK. Sector Plan for Environment, Water, Sanitation and Regional Development. Nairobi, Kenya: Government of the Republic of Kenya; 2018.
7. MoH. Prototype County Environmental Health and Sanitation Bill. Nairobi, Kenya: Ministry of Health; 2016.
8. MoH. National ODF Kenya 2020 Campaign Framework 2016/17-2019/20. Nairobi, Kenya: Ministry of Health; 2016.
9. MoWSI. Development of the National Sanitation Management Policy (NSMP) in Kenya. A narrative descriptive account of the journey. In: Ministry of Water SaI, editor. Nairobi, Kenya: Republic of Kenya; 2022.
10. WASREB. Guidelines on Sanitation Levy And Trade Effluent Surcharge by Water Service Providers in Kenya. Nairobi, Kenya: Water Services Regulatory Board; 2019.
11. WASREB. Guideline for provision of water and sanitation services in rural and underserved areas. Nairobi, Kenya: Water Services Regulatory Board; 2019.
12. WASREB. IMPACT 15. A Performance Report of Kenya's Water Services Sector - 2021/22. Nairobi, Kenya: Water Services Regulatory Board; 2023.
13. NCCA. The Report of the Sectoral Committee on Water And Sanitation on the Consideration Of Sessional Paper No. 3 Of 2018 on the Water and Sanitation Services Policy. Nairobi, Kenya: Nairobi City County Assembly; 2019.
14. NCWSC. Strategic Plan 2019/20-2023/24. Nairobi, Kenya: Nairobi City Water and Sewerage Company Limited; 2019.
15. AWSB. Strategic Guidelines for Improving Water and Sanitation Services in Nairobi's Informal Settlements. Nairobi, Kenya: Athi Water Service Board; 2009.
16. AWWDA. Strategic Plan 2018-2022. Nairobi, Kenya: Athi Water Works Development Agency; 2018.
17. GoK. Constitution of Kenya. Nairobi, Kenya: Government of Kenya; 2010.
18. GoK. Third Medium Term Plan 2018 – 2022: Transforming Lives: Advancing socio-economic development through the "Big Four": Government of the Republic of Kenya; 2018.
19. NCC. County Integrated Development Plan 2023-2027. In: County NC, editor. Nairobi, Kenya: Nairobi City County; 2023.
20. County Annual Development Plan (CADP) 2022/23, (2021).
21. Integrated Urban Development Master Plan for the City of Nairobi, (2014).
