# Supplementary material for: Business-as-usual and fantasy planning – an analysis of equity within climate adaptation planning for sanitation in Nairobi
Source: PLoS One. 2025 Dec 30;20(12):e0339272. doi: 10.1371/journal.pone.0339272 (PMC12752985; doi:10.1371/journal.pone.0339272)
Supplement: S3 Table — (PDF) [file pone.0339272.s003.pdf]

SUPPLEMENTAL INFORMATION: Business-as-usual and fantasy planning – an analysis of equity withing climate adaptation planning in Nairobi

S3 Table. *Summary of reviewed sanitation investments in Nairobi county with focus on specific references to sanitation system adaptation and equity considerations*

Investment Title (status)	Details on investment, relevance for service regimes, climate adaptation relevance	Main findings and remarks on equity considerations
AfDB Feasibility Studies and Detailed Designs for the Nairobi Inclusive Sanitation Improvement Project (ongoing) *(1)*	Feasibility study for enhanced sewerage and faecal sludge management in Nairobi<br>*Acknowledges impact of flooding on sanitation*<br>**covers all service regimes**	*Distribution:* Adheres to City Wide Inclusive Sanitation (CWIS) principles for sustainable FSM and sewage infrastructure across the value chain<br>*Recognition:* Proposes subsidized sewer connections in low-income urban areas.
GCF Infrastructure Climate Resilient Fund (ICRF) (approved)[1] *(2)*	Includes investments for rehabilitating roads and bridges in Nairobi county; indirectly affects sanitation services reliant on road-based transport.<br>*Climate resilience focus but not sanitation specific*<br>Indirect impact on: ***ST/RT, HT/RT, (ST/NC; HT/NC)***	*unspecific*
AfDB/AFD Nairobi Rivers Basin Rehabilitation and Restoration Program : Sewerage Improvement Project -Phase II (ongoing, 2019 - 2024) *(3)*	Expansion of sewer network and construction of faecal sludge disposal facilities and ablution blocks; rehabilitation of Dandora WWTP; includes capacity development for improved FSM<br>*Sewer design to take climate scenarios in consideration*<br>**PT; ST/SEWER; HT/SEWER; ST/RT; HT/RT**	*Distribution:* Concentrates on sewered sanitation, but includes delivery in informal areas and simplified sewers.<br>*Recognition:* Acknowledges the need for improved sanitation in informal areas.
WB Kenya Urban Support Program (ongoing 2017 – 2023) *(4)*[1]	Focuses on urban-level capacity building and infrastructure, including urban drainage systems; water and sanitation investments are not included in the eligible investment menu<br>Few references to climate change in relation to flood resilience<br>Indirect impact: Potentially benefits **all service regimes** by reducing flood risk.	*unspecific*
WB Second Kenya Informal Settlements Improvement Project (KISIP2) (ongoing 2021 – 2025) *(5, 6)*[1]	Targets settlement-level planning, tenure security, infrastructure upgrading including improvements of drainage; support to utilities to scale-up water and sanitation in informal settlements.<br>*Acknowledges that climate resilience is not centre of investment design but infrastructure should adhere to climate resilience standards*	*Distribution:* Indicators only relates to sewerage connections but there is precedent for inclusion of non-sewered sanitation from Kilifi County<br>*Recognition:* Identifies access challenges in urban informal settlements.<br>*Procedures:* Aims to improve tenure security to facilitate access to basic services

	**PT, ST/SEWER, ST/RT, ST/NC**	
Project information Athi Water Works Development Agency *(7)*[2]	Mainly construction and rehabilitation of trunk and reticulation sewers in various areas including low-income areas; includes rehabilitation of Kariobangi WWTP and construction stabilisation ponds, includes construction of public toilet blocks in Kibera<br>*No specific references to climate change*<br>**PT, ST/SEWER, HT/SEWER**	*unspecific*

[1] No specific information on implementation status and specific measures in Nairobi available

[2] Partly conflicting information about implementation status of investments

COPING (NC/NT) = People don't have access to toilets us coping mechanisms; PUBLIC TOILET = People rely on public toilets as main form of sanitation; ST/NC = Shared toilets that are NOT adequately managed; CONTAINER (ST/RT) = Shared toilets that are regularly emptied; ST/SEWER = Shared toilets connected to the sewer system; HT/NC = Household that are not adequately managed; HT/RT = Household toilets that regularly emptied; HT/SEWER = Household toilets connected to the sewer system

References:

1. AWF. Nairobi Inclusive Sanitation Improvement Project. Appraisal Report. Abidijan, Cote d'Ivoire: African Water Facility (AWF); 2019.
2. GCF. Funding Proposal: FP205: Infrastructure Climate Resilient Fund (ICRF). Green Climate Fund; 2023.
3. AfDB. Nairobi Rivers Basin Rehabilitation And Restoration Program: Sewerage Improvement Project Phase II. Project Appraisal Report. African Development Bank (AfDB); 2018.
4. The World Bank. Kenya - Urban Support Program: Project Appraisal Document. Washington DC: The World Bank; 2017.
5. The World Bank. Kenya - Second Kenya Informal Settlements Improvement Project: Project Appraisal Report. Washington DC: The World Bank; 2020.
6. Kamunyori S. Disclosable Version of the ISR - Second Kenya Informal Settlements Improvement Project - P167814 - Sequence No : 06 Washington DC: The World Bank; 2023.
7. AWWDA. Our Projects: Nairobi County: Athi Water Works Development Agency (AWWDA); 2023 [Available from: https://www.awwda.go.ke/nairobi-county/.
