# Supplementary material for: Business-as-usual and fantasy planning – an analysis of equity within climate adaptation planning for sanitation in Nairobi
Source: PLoS One. 2025 Dec 30;20(12):e0339272. doi: 10.1371/journal.pone.0339272 (PMC12752985; doi:10.1371/journal.pone.0339272)
Supplement: S4 File — (PDF) [file pone.0339272.s004.pdf]

SUPPLEMENTAL INFORMATION: Business-as-usual and fantasy planning – an analysis of equity withing climate adaptation planning in Nairobi

S4 Annex. Description of population forecasting method using ArcGIS Pro time series forecasting tools

In order to quantify the distribution of people using the different sanitation service regimes for Nairobi for the year 2030 we followed the steps below.

1. **Historic population time series data preparation (2000 -2022)**
   a. *Population Data Acquisition:* We obtained population raster data spanning from 2000 to 2022 from WorldPop (1)
   b. *Data integration:* Using ArcGIS Pro, we integrated the raster data with our SaniRegime_2030 model to produce a comprehensive attribute table containing population figures for the specified period
   c. *Outlier identification and removal:* We exported the attribute table to MS Excel and employed the Standard Deviation Method to cleanse the dataset of outliers. By calculating the mean (M) and standard deviation (σ) for each polygon, data points lying outside the M±3σ range were flagged as outliers and excluded, ensuring the reliability of our time series data.

2. **Population forecasting with ArcGIS Pro**
   a. Forecasting setup: We input the refined time series data into ArcGIS Pro and utilised the space-time cube tool to project the 2030 population figures, guided by the steps outlined by Li, Liu (2)
   b. *Forecasting methods:* We generated forecasts using Curve Fit, Exponential Smoothing, and Forest-based models, deliberately omitting three time steps for validation.
   c. Forecast evaluation: The "Evaluate Forecasts by Location" geoprocessing tool was employed to ascertain the most accurate forecasting method for each polygon, ensuring the selection of the most fitting projection model (2).

3. **Data cleaning and validation**
   a. Preliminary cleaning: After exporting the forecast data to Excel, we conducted a thorough examination for outliers and illogical values, such as negative figures, applying specific rules for manually cleaning the data:
      i. *Elimination of Negative Growth:* Acknowledging past population trends (Figure 1) and external growth forecasts (3, 4), we presupposed a non-negative growth trajectory. Any forecast suggesting a decrease from the 2022 population level was adjusted to reflect a static population, thereby also eliminating negative values.

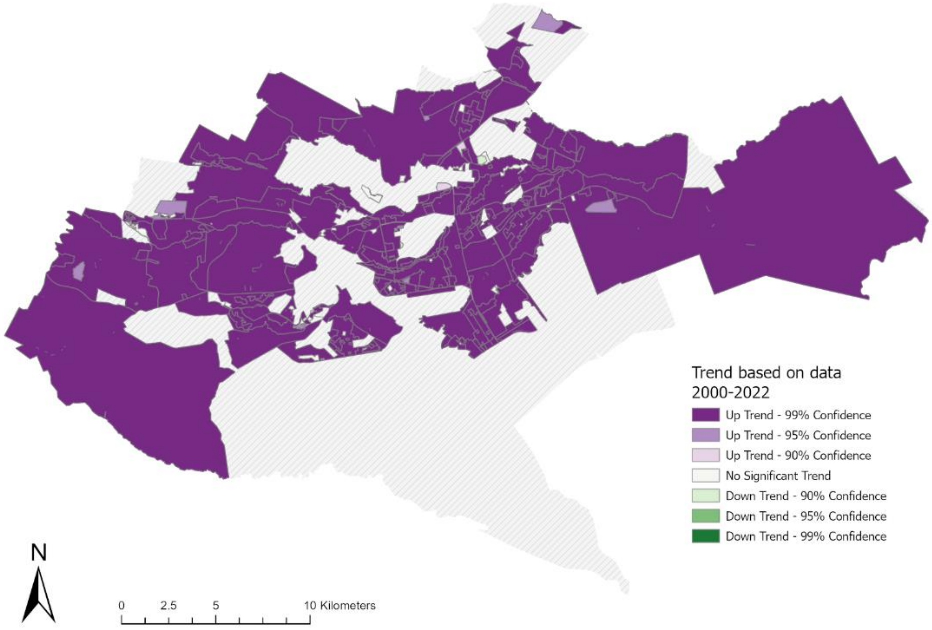

Figure 1: Growth for sub-areas based on historic population data (2000-2022)

ii. *Adjustment of unrealistic forecasts:* Given the relatively short historic time series the extrapolation result showed sensitivity to the number of time steps excluded for validation (the tool usually is set to exclude 10% of the totally observed time steps by default and 25% of the time steps is the maximum). We explored a range of time step exclusion scenarios ranging from 0 (no validation) to 5 (maximum possible value). We then evaluated the mean (M) and median (Mdn) forecasted population for 2030 across these scenarios. For all polygons where M/Mdn=1±0.15 we manually checked the extrapolation curve fit manually adjusting forecasts that deviated significantly from realistic expectations often associated with exponential growth curves (see screenshot of example below). We either adjusted value to next best fitting curve or in case none of the suggested extrapolation curves seemed fit we assumed static population.

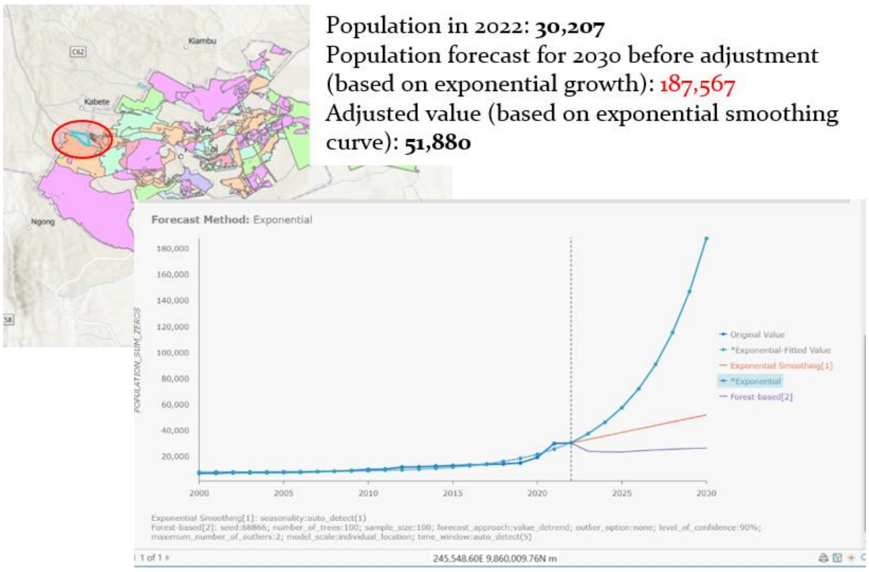

Figure 2: Example of unrealistic forecast value that was adjusted

After data cleaning as described above our total population forecast for Nairobi 2030 changed from 5.5 million (raw data) to 6.1 million cleaned data.

4. Visual summaries

Figure 3 illustrates the forecasting methods selected post-cleaning for each sub-area.

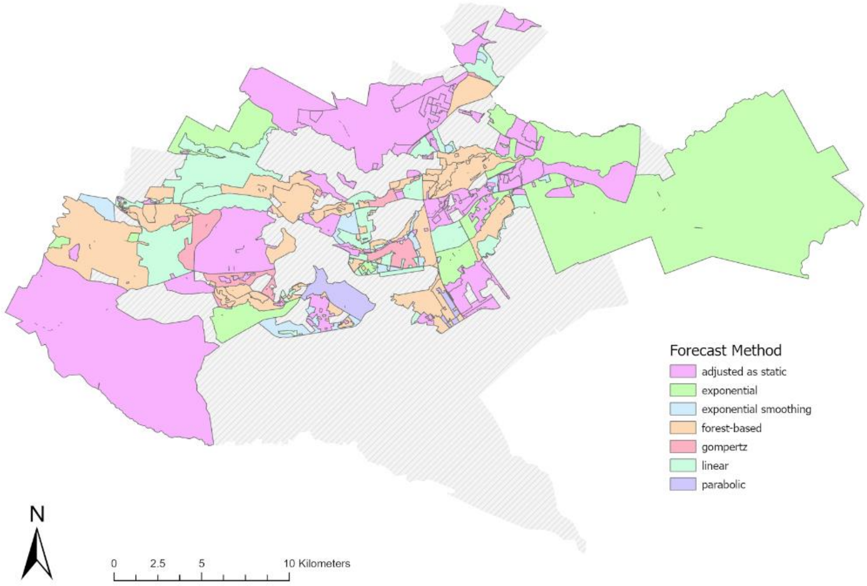

Figure 3: Overview of forecasting methods selected post-cleaning

Figure 4 depicts the population numbers for each sub-area following data cleansing

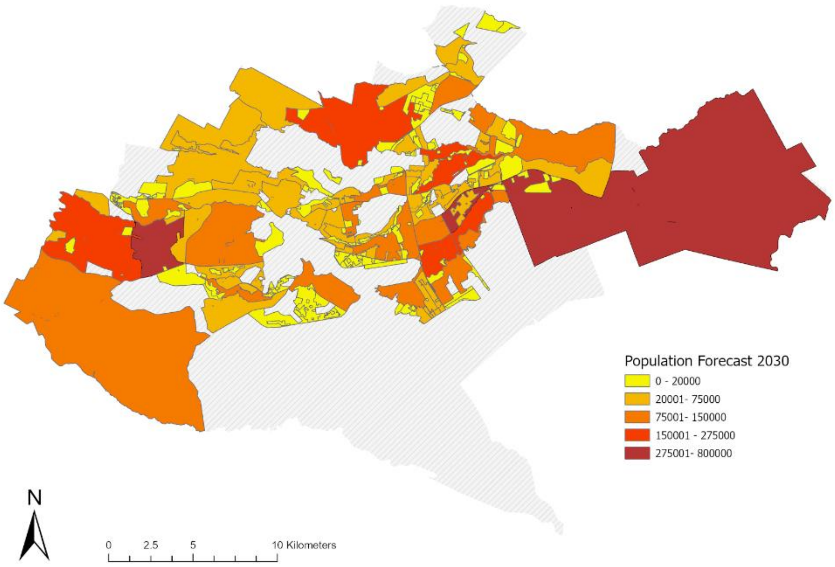

Figure 4: Population numbers for each sub-area following data cleansing

References

1. WorldPop. WorldPop Hub: Population Counts Southampton, UK: University of Southampton; 2024 [Available from: https://hub.worldpop.org/.
2. Li S, Liu J, Butler K. ArcGIS Blog [Internet]: ESRI. 2021. [cited 2024]. Available from: https://www.esri.com/arcgis-blog/products/arcgis-pro/analytics/looking-to-the-future-using-gis-to-model-and-predict-population/.
3. U.S. Census Bureau. Kenya Annual Five-Year Age Group Population Estimates by Sex for 2015 to 2030: National, and First- and Second-Order Administrative Divisions. In: U.S. Census Bureau PD, editor. 2023.
4. The World Bank. Kenya Urban Support Program 2023 [cited 2023. Available from: https://projects.worldbank.org/en/projects-operations/project-detail/P156777.
