# Supplementary material for: Business-as-usual and fantasy planning – an analysis of equity within climate adaptation planning for sanitation in Nairobi
Source: PLoS One. 2025 Dec 30;20(12):e0339272. doi: 10.1371/journal.pone.0339272 (PMC12752985; doi:10.1371/journal.pone.0339272)
Supplement: Attachment — Submitted filename: PONE-D-24-10746_reviewer.pdf [file pone.0339272.s005.pdf]

PLOS ONE

Business-as-usual and fantasy planning – an analysis of equity within climate adaptation planning for sanitation in Nairobi

--Manuscript Draft--

**Manuscript Number:**	PONE-D-24-10746
**Article Type:**	Research Article
**Full Title:**	Business-as-usual and fantasy planning – an analysis of equity within climate adaptation planning for sanitation in Nairobi
**Short Title:**	Equity within climate adaptation planning for sanitation
**Corresponding Author:**	Leonie K. Hyde-Smith, MSc<br>University of Leeds Faculty of Engineering and Physical Sciences<br>Leeds, UNITED KINGDOM
**Keywords:**	sanitation; climate change; equity; urban planning; wastewater management; sewerage; SDGs; urban areas; adaptation
**Abstract:**	This paper explores the disconnect between policy rhetoric and implementation at the intersection of sanitation equity and climate change in Nairobi, Kenya. To examine the current sanitation adaptation trajectory, we reviewed Nairobi's sanitation policies, planning, and investment frameworks, focusing on their integration with climate adaptation strategies and consideration of equity in terms of distribution, recognition and processes. We used a socio-technical regime framework to map the current sanitation service configurations in Nairobi and projected their future under different climate change scenarios.<br>Our findings reveal a disconnection between policy rhetoric and implementation, prioritising sewerage development at the expense of other sanitation regimes. Despite recognising equity issues in policy, substantive action towards equitable sanitation governance is lacking. The anticipated impact of climate change on Nairobi's sanitation sector suggests an exacerbation of existing service inequalities. Our projection indicates that by 2030, a sizeable portion of Nairobi's residents will experience poor sanitation services.<br>Our study emphasizes the critical need for a fundamental paradigm shift. It calls for an earnest discussion on delivering high-quality, resilient sanitation services at scale including both sewer and non-sewered sanitation and necessitating substantial public investment and support for all systems. This reappraisal is imperative not only for meeting the Sustainable Development Goals but for ensuring equitable and sustainable sanitation solutions in the face of climate change.
**Order of Authors:**	Leonie K. Hyde-Smith, MSc
	Daniel Ddiba, PhD
	Sarah Dickin, PhD
	Domenic Kiogora, MSc
	Anna L. Mdee
	Vanessa Reinfelder
	Katy E. Roelich, PhD
	Barbara E. Evans
**Additional Information:**	
**Question**	**Response**
**Financial Disclosure**<br><br>Enter a financial disclosure statement that describes the sources of funding for the work included in this submission. Review	Yes

Powered by Editorial Manager® and ProduXion Manager® from Aries Systems Corporation

the submission guidelines for detailed requirements. View published research articles from *PLOS ONE* for specific examples.<br><br>This statement is required for submission and **will appear in the published article** if the submission is accepted. Please make sure it is accurate.<br><br>**Funded studies**<br>Enter a statement with the following details:<br>• Initials of the authors who received each award<br>• Grant numbers awarded to each author<br>• The full name of each funder<br>• URL of each funder website<br>• Did the sponsors or funders play any role in the study design, data collection and analysis, decision to publish, or preparation of the manuscript?<br><br>Did you receive funding for this work?	
Please add funding details.<br>as follow-up to "**Financial Disclosure**<br><br>Enter a financial disclosure statement that describes the sources of funding for the work included in this submission. Review the submission guidelines for detailed requirements. View published research articles from *PLOS ONE* for specific examples.<br><br>This statement is required for submission and **will appear in the published article** if the submission is accepted. Please make sure it is accurate.	This work was supported by the UKRI Engineering and Physical Science Research Council (EPSRC) through a PhD studentship received by the first author (L.H.-S.) as part of the EPSRC Centre for Doctoral Training in Water and Waste Infrastructure and Services Engineered for Resilience (Water-WISER). EPSRC Grant number: EP/S022066/1

Powered by Editorial Manager® and ProduXion Manager® from Aries Systems Corporation

**Funded studies**<br>Enter a statement with the following details:<br>• Initials of the authors who received each award<br>• Grant numbers awarded to each author<br>• The full name of each funder<br>• URL of each funder website<br>• Did the sponsors or funders play any role in the study design, data collection and analysis, decision to publish, or preparation of the manuscript?<br><br>Did you receive funding for this work?"	
Please select the country of your main research funder (please select carefully as in some cases this is used in fee calculation).<br>as follow-up to "**Financial Disclosure**<br><br>Enter a financial disclosure statement that describes the sources of funding for the work included in this submission. Review the submission guidelines for detailed requirements. View published research articles from *PLOS ONE* for specific examples.<br><br>This statement is required for submission and **will appear in the published article** if the submission is accepted. Please make sure it is accurate.<br><br>**Funded studies**<br>Enter a statement with the following details:<br>• Initials of the authors who received each award<br>• Grant numbers awarded to each author<br>• The full name of each funder<br>• URL of each funder website<br>• Did the sponsors or funders play any role in the study design, data collection and analysis, decision to publish, or preparation of the manuscript?<br><br>Did you receive funding for this work?"	UNITED KINGDOM - GB
**Competing Interests**	The authors have declared that no competing interests exist.

Use the instructions below to enter a competing interest statement for this submission. On behalf of all authors, disclose any competing interests that could be perceived to bias this work—acknowledging all financial support and any other relevant financial or non-financial competing interests.<br><br>This statement is required for submission and **will appear in the published article** if the submission is accepted. Please make sure it is accurate and that any funding sources listed in your Funding Information later in the submission form are also declared in your Financial Disclosure statement.<br><br>View published research articles from *PLOS ONE* for specific examples.<br><br>**NO authors have competing interests**<br><br>Enter: *The authors have declared that no competing interests exist.*<br><br>**Authors with competing interests**<br><br>Enter competing interest details beginning with this statement:<br><br>*I have read the journal's policy and the authors of this manuscript have the following competing interests: [insert competing interests here]*<br><br>* typeset	
**Ethics Statement**<br><br>Enter an ethics statement for this submission. This statement is required if the study involved:<br><br>• Human participants<br>• Human specimens or tissue<br>• Vertebrate animals or cephalopods<br>• Vertebrate embryos or tissues	N/A

Powered by Editorial Manager® and ProduXion Manager® from Aries Systems Corporation

• Field research<br><br>Write "N/A" if the submission does not require an ethics statement.<br><br>General guidance is provided below. Consult the submission guidelines for detailed instructions. **Make sure that all information entered here is included in the Methods section of the manuscript.**	

**Format for specific study types**<br><br>**Human Subject Research (involving human participants and/or tissue)**<br>• Give the name of the institutional review board or ethics committee that approved the study<br>• Include the approval number and/or a statement indicating approval of this research<br>• Indicate the form of consent obtained (written/oral) or the reason that consent was not obtained (e.g. the data were analyzed anonymously)<br><br>**Animal Research (involving vertebrate animals, embryos or tissues)**<br>• Provide the name of the Institutional Animal Care and Use Committee (IACUC) or other relevant ethics board that reviewed the study protocol, and indicate whether they approved this research or granted a formal waiver of ethical approval<br>• Include an approval number if one was obtained<br>• If the study involved *non-human primates*, add *additional details* about animal welfare and steps taken to ameliorate suffering<br>• If anesthesia, euthanasia, or any kind of animal sacrifice is part of the study, include briefly which substances and/or methods were applied<br><br>**Field Research**<br><br>Include the following details if this study involves the collection of plant, animal, or other materials from a natural setting:<br>• Field permit number<br>• Name of the institution or relevant body that granted permission	
**Data Availability**<br><br>Authors are required to make all data underlying the findings described fully available, without restriction, and from the time of publication. PLOS allows rare exceptions to address legal and ethical concerns. See the PLOS Data Policy and FAQ for detailed information.	Yes - all data are fully available without restriction

A Data Availability Statement describing where the data can be found is required at submission. Your answers to this question constitute the Data Availability Statement and **will be published in the article**, if accepted.<br><br>**Important:** Stating 'data available on request from the author' is not sufficient. If your data are only available upon request, select 'No' for the first question and explain your exceptional situation in the text box.<br><br>Do the authors confirm that all data underlying the findings described in their manuscript are fully available without restriction?	
**Describe where the data may be found in full sentences. If you are copying our sample text, replace any instances of XXX with the appropriate details.**<br><br>• If the data are **held or will be held in a public repository**, include URLs, accession numbers or DOIs. If this information will only be available after acceptance, indicate this by ticking the box below. For example: *All XXX files are available from the XXX database (accession number(s) XXX, XXX.).*<br>• If the data are all contained **within the manuscript and/or Supporting Information files**, enter the following: *All relevant data are within the manuscript and its Supporting Information files.*<br>• If neither of these applies but you are able to provide **details of access elsewhere**, with or without limitations, please do so. For example:<br><br>*Data cannot be shared publicly because of [XXX]. Data are available from the XXX Institutional Data Access / Ethics Committee (contact via XXX) for researchers who meet the criteria for access to confidential data.*<br><br>*The data underlying the results presented in the study are available from (include the name of the third party*	All relevant data are within the manuscript and its Supporting Information files.

and contact information or URL).<br>• This text is appropriate if the data are owned by a third party and authors do not have permission to share the data.<br><br>* typeset	
Additional data availability information:	Tick here if the URLs/accession numbers/DOIs will be available only after acceptance of the manuscript for publication so that we can ensure their inclusion before publication.

Powered by Editorial Manager® and ProduXion Manager® from Aries Systems Corporation

Manuscript
Click here to access/download;Manuscript;CC Sanitation Equity Nairobi.docx

Business-as-usual and fantasy planning – an analysis of equity within climate adaptation planning for sanitation in Nairobi

Leonie K. Hyde-Smith[1]*, Daniel Ddiba[2], Sarah Dickin[3], Domenic Kiogora[4], Anna L. Mdee[5], Vanessa Reinfelder[6], Katy E. Roelich[7], and Barbara E. Evans[1]

[1] School of Civil Engineering, University of Leeds, Leeds, United Kingdom

[2]Stockholm Environment Institute, Stockholm, Sweden

[3] Swedesd - Sustainability Learning and Research Centre, Department of Women's and Children's Health, Uppsala University, Sweden

[4]Meru University of Science and Technology, Department of Biological Sciences, Meru, Kenya

[5] University of Leeds School of Politics and International Studies, Leeds, United Kingdom

[6] gruppe F, Berlin, Germany

[7] University of Leeds School of Earth and Environment / Sustainability Research Institute, Leeds, United Kingdom

*Corresonding author

Email: cnlkhs@leeds.ac.uk

Abstract

This paper explores the disconnect between policy rhetoric and implementation at the intersection of sanitation equity and climate change in Nairobi, Kenya. To examine the current sanitation adaptation trajectory, we reviewed Nairobi's sanitation policies, planning, and investment frameworks, focusing on their integration with climate adaptation strategies and consideration of equity in terms of distribution, recognition and processes. We used a socio-technical regime framework to map the current sanitation service configurations in Nairobi and projected their future under different climate change scenarios.

Our findings reveal a disconnection between policy rhetoric and implementation, prioritising sewerage development at the expense of other sanitation regimes. Despite recognising equity issues in policy, substantive action towards equitable sanitation governance is lacking. The anticipated impact of climate change on Nairobi's sanitation sector suggests an exacerbation of existing service inequalities. Our projection indicates that by 2030, a sizeable portion of Nairobi's residents will experience poor sanitation services.

Our study emphasizes the critical need for a fundamental paradigm shift. It calls for an earnest discussion on delivering high-quality, resilient sanitation services at scale including both sewer and non-sewered sanitation and necessitating substantial public investment and support for <u>all</u> systems. This reappraisal is imperative not only for meeting the Sustainable Development Goals but for ensuring equitable and sustainable sanitation solutions in the face of climate change.

Background

2023 marked the temporal halfway point in the implementation of the Sustainable Development Goals (SDGs), including target 6.2 on achieving access to adequate and equitable sanitation for all. Recent WHO and UNICEF Joint Monitoring Programme (JMP) data shows increased basic sanitation coverage between 2015 and 2022. However, at the current rate of improvement, none of the SDG regions are on track to achieve universal access by 2030 (1). Inequitable access to sanitation service provision is still prevalent globally (1-3) and gained heightened attention during the Covid-19 response (4-6). However, sanitation investments and funding arrangements are biased towards subsidising capital investment

and operational costs of centralised sewerage and treatment systems, which often primarily tend to more affluent parts of the population (7-10).

The 6th Intergovernmental Panel on Climate Change (IPCC) Assessment Report has highlighted that climate change amplifies inequalities and undermines sustainable development across regions (11, p.1174); amongst others, climate change increases the risks of urban sanitation failures (12). There is growing recognition that climate-adaptive sanitation infrastructure is non-negotiable for avoiding the risk of reversing progress towards universal access to safe sanitation services (13, 14) and thereby worsening inequalities. However, attention to adaptation needs within urban sanitation systems has been limited and primarily focused on technology solutions (15). Sanitation adaptation is mainly happening in high-income countries, focusing on retrofitting and upgrading old infrastructure approaching the end of its design life (16, 17).

Equity considerations in climate change adaptation efforts are generally assessed through a justice framework (18). The commonly adopted conceptualisations of climate (adaptation) justice are mainly derived from the environmental justice scholarship (19, 20) and are frequently composed of multiple components such as distributive, procedural and recognition justice (21-23). A global systematic review of peer-reviewed empirical research on adaptation responses (18) found that around 60% of the 1,682 reviewed adaptation responses considered social equity in terms of distributive and procedural justice aspects. However, unlike the more advanced scholarship on just transitions for other urban sectors such as energy (21, 22, 24, 25) or mobility (26, 27) justice aspects on the interface between urban sanitation planning and climate change adaptation are relatively underexplored.

Against this backdrop, this study explores if and how sanitation equity aspects are considered in sanitation adaptation planning in a city with an existing sanitation service gap and service inequalities.

We selected the example of Nairobi, Kenya, since there is good data availability on the sanitation sector of Nairobi (28-30) and Kenya (31). Van Welie et. al (28) extend the scholarship of socio-technical regimes to adapt the socio-technical regime perspective to complex and heterogeneous basic service sectors such as sanitation service provision in many low- and middle-income country cities where sanitation systems consist of multiple, diverse sub-systems of different grades of formality.

Consequently, they distinguish the two levels of *sectoral regime* and *service regimes*. The sectoral regime encompasses various sanitation service infrastructures and organisational arrangements, while service regimes describe the diverse ways in which human waste is managed in a city. This framework highlights the degrees of alignment or misalignment among service regimes, affecting the efficiency and inclusiveness of services. It is generally presumed that aligned service regimes work well together, create efficient and complementary services across various providers and are supported by suitable regulations and policies. On the other hand, misaligned regimes lead to inefficient services and a lack of recognition for different service approaches within a sector (28, 32). Building upon this conceptualisation we explored Nairobi's sanitation landscape. Nairobi's sanitation sector is classified as a 'splintered regime', marked by heterogeneity and disjointed coexisting service regimes, particularly in non-sewered areas (30, 33). Such splintered regimes often give rise to informal ad-hoc coping strategies such as 'flying toilets' in response to misaligned services (28).

The distinctive configuration of Nairobi's sanitation services is the result of its unique historical, cultural, and economic contexts (32, 34-36), necessitating a nuanced understanding of these factors. However, there are limited studies on the equity of water and sanitation services in Nairobi (28, 29, 37). Throughout the last two decades, Kenya's water sector has (at least rhetorically) proclaimed the intention to engage with low-income groups through a 'pro-poor' or 'inclusive' service approach. Moreover, the Kenyan Constitution recognises the human right to sanitation, although there is little evidence that formal inclusion in the constitution has led to improved service delivery (38). Furthermore, the recent hosting of the inaugural African Climate Summit in Nairobi underscores a strong political extraversion to signal to international audiences the dedication to climate change adaptation and mitigation policy, even though our study points to discrepancies between these proclaimed commitments and actual implementation.

Our analysis follows several objectives:

1. Characterise the current sanitation service configuration in Nairobi and inherent sanitation service inequalities from a distributive justice perspective
2. Evaluate how the different sanitation service configurations are likely to be affected by climate change

3. Assess if and how climate adaptation is considered in the current sanitation planning and investment framework of the city, and vice versa, if sanitation is considered within the broader climate adaptation planning and investment framework of Nairobi
4. Estimate how the effects of climate change are likely to alter existing service inequalities, considering the current sanitation adaptation planning framework.

Methods

Visualisation and quantification of current sanitation service configurations in Nairobi.

We examined the unequal distribution of sanitation services across Nairobi, employing a socio-technical regime framework.

To understand the current layout of sanitation services in Nairobi County, we used georeferenced data (shapefiles showing land use and income classes) from previous studies on water inequality in Nairobi (37, 39) and cross-referenced these with recent satellite imagery (40) validating currency and accuracy. Given the rapid urbanisation and population growth in Nairobi, the most recent classifications of income-specific residential and non-residential areas (37) were used.

We overlaid a map of the current and planned sewer network from the Nairobi Masterplan and combined this data with past analysis of sanitation coverage and residential typology (39), alongside current information on sanitation gathered from various documents and stakeholders in Nairobi. Validating this data with the recent basemap, we conducted a geospatial analysis to map the main types of sanitation systems in Nairobi following the allocation process described in Figure 1.

Figure 1: Description of sanitation regime mapping process (current situation)

To estimate the number of people served by each type of sanitation system, we overlaid the shapefiles with updated gridded population data for 2022 (41) produced by the WorldPop Research Group at the University of Southampton.WorldPop derive population data from projections using the 2019 Kenyan

population and housing census as baseline data. Aggregate population counts for census areas are re-distributed within each boundary onto 100 by 100m grid cells.

As shown in Figure 1 we followed an iterative approach and validated and refined the distribution from our geospatial analysis with results from relevant studies (42-44), sector publications (45) and information provided by sanitation stakeholders in Nairobi (46).

Climate change impacts on current sanitation service configuration in Nairobi

As a next step, we combined descriptive climate scenarios for East Africa (47, 48) and results of a recent systematic review on climate impacts on urban sanitation systems (12) to generally describe the likely impacts of climate change on the different sanitation service regimes in Nairobi. Acknowledging the uncertainty surrounding Kenya's climate trajectory in the coming decades, we considered the effect of different climate scenarios. Under the Future Climate for Africa research, the HyCRISTAL project delineated three basic scenarios of how climate change could be felt in urban East Africa by 2050 (48): i) *Much wetter, large increase in heavy rainfall and hotter*; ii) *Increase in extreme rainfall and hotter;* iii) *Much hotter and drier with more erratic rainy seasons*. Table 1 provides a more detailed summary of the scenarios/futures.

Table 1: Summary of 2050 climate scenarios for East Africa according to HyCRISTAL research

	Future 1: Much wetter, large increase in heavy rainfall and hotter	*Future 2: Increase in extreme rainfall and hotter*	*Future 3: Much hotter and drier with more erratic rainy seasons*
Description	Increased rainfall during both long and short rainy seasons; More intense rainfall and increased frequency of storms; Average annual temperature increases of about 2°C and hotter temperature extremes.	No substantial changes in total rainfall amounts but more intense rainfall events and more frequent occurrence of extreme storms, longer dry spells, Average annual temperature increase of about 2-3°C and hotter temperature extremes.	Substantial increase of average annual temperatures (by about 3°C) and more frequent occurrence of extreme heat.<br>Decrease in total annual rainfall amount and increase duration of dry spells and more frequent occurrence of drought events; Occasional occurrence of extreme rainfall and storms
Most critical climate change effect on the sanitation system	More frequent occurrence of recurrent and rapid onset floods (flash floods)	More frequent occurrence of flash floods	Water shortages;<br>Occasional occurrence of flash floods

Source: Summary based on (47)

We attempted to map and estimate the present and future flood risk in Nairobi by overlaying our geospatial sanitation service regime analysis with flood model data (pluvial and fluvial) for Nairobi (49, 50). We compared various flood return periods (1-in-10, 1-in-50 and 1-in-100-year) for the present (2020 data) and projections for the years 2030 and 2050. Given the short time frame, we chose the moderate 'middle of the road' shared-socioeconomic pathway' scenario (SSP2-4.5) estimates. However, comparing flood depth and extent for all return-period scenarios between 2020, 2030, and even 2050 data (defended, 50-percentile) showed only minor changes. Given the unsubstantial modelled modifications combined with the relatively coarse granularity of the gridded population data (100m x 100m cells) and general limitations of estimating flood exposure using gridded population datasets (51), we refrained from quantifying the changes in population at risk.

Analysis of policy and planning framework

To gauge the current trajectory of climate adaptation and sanitation, we conducted a comprehensive document review of relevant (national and county-level) laws, policies, strategies, and planning documents that are likely to shape future sanitation (adaptation) planning in Nairobi. Apart from sanitation (or water sector) specific documents, we also included general framework documents such as the Constitution of Kenya and the Vision 2030 national development blueprint, as well as documents guiding the country's and county's approach to climate adaptation (e.g., the Climate Change Act and the Nairobi City County Climate Change Action Plan). We also reviewed available information on current and planned investments into Nairobi's sanitation sector, considering the strategic planning and budget documents of Nairobi City County (NCC), Nairobi City Water and Sewerage Company (NCWSC) and Athi Water Works Development Agency (AWWDA) and ongoing and planned investment of development partners (many of which are captured in NCC, NCWSC or AWWDA planning). We used the SEI Aid Atlas (52) as a starting point to identify relevant ongoing or planned investments made by various donors to Kenya. We subsequently examined detailed investment information on the websites of the specific donors and relevant investment databases African Water Facility (AWF), African Development Bank (AfDB) and World Bank (WB). We validated our selection with information from the Nairobi Integrated Urban Development Master Plan (53) and NCWSC, Water Sector Trust Fund (WSTF) representatives and non-public actors such as Sanergy and WSUP.

So far, no coherent definition for 'just' or 'equitable' climate adaptation of urban sanitation has been proposed. We, therefore, drew upon environmental and energy justice scholarship and propose the following key aspects of 'equitable' climate adaptation of urban sanitation (Table 2). We applied these evaluation criteria for deductive coding in NVivo across all information on sanitation planning, investments and climate adaptation.

Table 2 Aspects of equitable sanitation adaptation (based on Wood and Roelich (22))

Tenets	*Evaluation criteria relevant for equitable sanitation adaptation*
Distribution<br><br>*How are the benefits and burdens of sanitation provision (or failures) distributed through space and time?*	Which sanitation service configurations are represented in the current or planned adaptation measures?<br>Who bears the environmental/public health risk before the investment/in case of failure of the measure?<br>Does the adaptation measure change the distribution of environmental/public health risks between the different service regimes?<br>Is there explicit recognition of aspects of intergeneration justice concerning environmental and public health risks?<br>Who bears the financial risk of the current or planned adaptation investment?<br>Is there explicit recognition of aspects of intergeneration justice concerning the financing mechanism?
Recognition<br><br>*How are population groups and individuals represented and recognised in reference to sanitation adaptation*	Do the urban sanitation (adaptation) initiatives acknowledge and prioritise the needs of marginalised and vulnerable population groups?<br>Do legal frameworks recognise and protect the rights of individuals and marginalised population groups to sanitation services, holding governments and institutions accountable?<br>Is there evidence whether the current or planned measure explicitly considers affordability for different population groups?
Procedures<br><br>*How are the processes involved in sanitation adaptation ameliorating injustices that have been identified*	Is there any evidence that the planning and decision-making process of the adaptation of the sanitation system considers structural inequalities in participation?<br>Do the planning and decision-making process include mechanisms for meaningful engagement with local populations, ensuring their needs and concerns are considered in sanitation adaptation planning?<br>Are reasons for inclusion/exclusion/prioritisation of adaptation measures concerning specific service configuration clear?

Estimate of Nairobi's sanitation future for 2030

Finally, we combine our different analysis streams to discuss plausible sanitation equity outcomes for sanitation provision in Nairobi 2030. We refer to our analysis of available policy, planning and investment documents to construct a sanitation service regime configuration for 2030 and estimate the sanitation equity implications, considering the likely impacts of climate change and planned adaptive measures to counter these. We use rasterised population data from WorldPop (2000-2022) to extrapolate the spatial distribution of Nairobi's population in 2030 (54). Acknowledging the different growth patterns in distinct parts of Nairobi, we used the ArcGIS Pro time series forecasting tools that

enabled us to evaluate different extrapolation methods against each other and choose the best-fit population trend for each sub-area by validation or overall fitness of observed values (55). We provide a detailed description of the forecasting method in the supplemental information (S4 Annex).

Results and Discussion

Nairobi's splintered sanitation sector regime

Urban sanitation transcends mere infrastructure. As explored by various scholars (e.g.,56, 57), the concept of socio-technical regimes provides a comprehensive framework for understanding how social practices, institutions, and technologies interact within a sector. In the context of the sanitation sector, socio-technical regimes offer a nuanced understanding of how sanitation systems are designed, maintained, and adapted over time, incorporating both technical and social dimensions.

Nairobi, a rapidly expanding city, grapples with significant urban disparities. The historical context of its urban water sector, marked by deliberate, uneven development during colonial times (36, 58) has left a complex legacy for post-independence water and sanitation planning. The city lacks an integrated system for managing and delivering sanitation services to the population. Instead, multiple actors and stakeholders provide sanitation services, often operating in isolation and frequently in some form of informality (33).

We use the conceptualisation of the sanitation service regime to unravel the unequal service distribution across Nairobi. We expand on Van Welie et al.'s (28) household-centric classification by incorporating neighbourhood outcomes (efficacy in excreta removal). Public and environmental health implications of sanitation occur in the household, neighbourhood, and broader cityscape (59). We argue for a comprehensive analysis of distributive equity in sanitation services, considering both individual service realities and broader public health implications, at least on a neighbourhood scale. We acknowledge falling short of covering city-scale sanitation outcomes, as we do not assess the effectiveness and adequacy of treatment and disposal. Importantly, our classification of 'good sanitation' (as represented in the matrix bottom right) is not to be equated with safely managed sanitation in terms of the SDG target 6.2 (Figure 2).

Figure 2: Matrix showing distributional equity implications of sanitation service regimes using Nairobi as an example

According to a detailed urban excreta flow assessment, only 34% of Nairobi's excreta is safely managed (42, 60), indicating substantial systemic failures across the sanitation service chain of all service regimes.

Residents lacking access to personal or communal toilets often resort to buckets, bags, or open defecation with (NT/NC or COPING regime), with public toilets (PUBLIC TOILET / PT regime) offering limited improvement from a public health perspective but failing to provide adequate individual sanitation. Furthermore, reliance on public toilets often coexists with other coping mechanisms, especially at night. In Nairobi, both options are associated with low-income, high-density slum settings.

Notably, many low-income areas, such as parts of Kibera or Mathare, are within NCWSC's service area but remain disconnected *ostensibly* due to costs, legal and infrastructural barriers, and inconsistent water supply.

Informal service providers organised in 'cartels' often control the water supply, sanitation emptying and solid waste collection in many informal settlements. The cartels control the pricing of basic services, restrict alternative service providers (including NCWSC staff) in their operations (through illegal charges and violence), intimidate residents, and are linked to deliberate acts of vandalism of sanitation infrastructure that threatens their business model (61-64). Most public toilets in low-income areas, often managed by community organisations, are connected to the sewer system but are poorly maintained, failing to provide adequate services (65).

Shared toilets (ST) on plots inhabited by tenants are probably the most common sanitation solution for low-income residents in Nairobi. These toilets are either connected to the sewer system (ST/SEWER) or have some form of underground storage (pit/tanks). Due to the rapid densification of the urban areas in Nairobi, most of the non-sewered facilities get emptied once full, mostly resulting in indiscriminate and unsafe disposal of faecal sludge (ST/NC regime), often in proximity to the point of emptying (65).
Shared sanitation is typically associated with high-density single-storey 'shacks' in low-income settlements, but as Mwau and Sverdlik (66) point out, as Nairobi densifies, low-income accommodation

is becoming increasingly heterogeneous and there has been a rise in high-rise (walk-up) low-quality tenement buildings with single-room rented accommodation and shared sanitation facilities. High-density tenement developments have put additional strain on the existing water and sewer systems and are frequently connected to informal sewers that drain raw sewerage into rivers such as the Mathare River (66).

Regular and formalised emptying for shared toilets is primarily provided for container-based sanitation systems run under the Fresh Life (FL) franchise, the non-profit entity of the international social enterprise Sanergy. Most FL toilets are shared by tenants of low-income housing plots and operated by the landlord (30). Toilets shared between more than two households are not counted as safely managed under the JMP SDG monitoring. However, scholars and practitioners have long highlighted the need to recognise well-managed shared toilets as an important intermediate step for reducing sanitation inequalities in dense urban settlements, albeit also acknowledging concerns about the lack of nuance in knowledge about the realities of 'shared access' (67-69). For simplification, we assume that currently, all toilets within the frequently emptied shared toilets regime in Nairobi are container-based systems operated by FL (CONTAINER).

We want to stress that inadequately managed road-based and poorly maintained sewer systems can pose a direct public health risk to urban residents. Nairobi's sewer system is partly dilapidated and lacks maintenance (63, 70). Sewer blockages, seepage and overflows distribute excreta within urban neighbourhoods with potentially similar adverse public health outcomes as overflowing pit latrines. In Nairobi, sewer seepage is mainly reported as a health risk in low-income areas (58). Still, there is relatively little data on the citywide distribution and extent of the problem.

The Nairobi City Water and Sewerage Company (NCWSC) provides water and sewerage services in Nairobi. Generally, household toilets connected to the sewer system (HT/SEWER) are associated with high-income or middle-income neighbourhoods (often apartment blocks) in the centre of the town. Yet, the city's sewer system, outdated and overburdened, struggles to keep pace with urban growth. The Dandora and Kariobangi wastewater treatment plants, operated by NCWSC, suffer from poor maintenance, leading to inefficient wastewater treatment and environmental pollution (71, 72).

Contrary to common belief, not all middle and high-income residences in Nairobi are connected to the sewer system. Many low-density affluent neighbourhoods, such as Karen or Muthaiga, are located outside the sewered area and rely on household toilets connected to underground tank systems (HT/RT). These toilets provide a good service for the individual and, due to the low density of the areas, are unlikely to pose a direct public health threat in the vicinity of use. However, we want to stress that they often do not meet the technical criteria of septic systems and are often served by informal exhauster services, largely controlled by cartels who frequently illegally dump their load into the environment (58, 61, 63). As such, these toilets might still be counted as not being safely managed according to the JMP criteria (73).

Spatial and quantitative distribution of sanitation services in Nairobi

Following the allocation process described in Figure 1 we arrived at an approximate current spatial distribution of sanitation service regimes, underscoring the complexity of addressing urban sanitation (Figure 3).

Figure 3: Approximate spatial distribution of dominant sanitation service regimes in Nairobi – present situation: COPING (NC/NT) = People don't have access to toilets us coping mechanisms; PUBLIC TOILET = People rely on public toilets as main form of sanitation; ST/NC = Shared toilets that are NOT adequately managed; CONTAINER (ST/RT) = Shared toilets that are regularly emptied; ST/SEWER = Shared toilets connected to the sewer system; HT/NC = Household that are not adequately managed; HT/RT = Household toilets that regularly emptied; HT/SEWER = Household toilets connected to the sewer system

Using population raster data, we estimated the approximate quantitative population distribution per service regime and validated our results against available sanitation data. We validated our data against multiple available sources and found that our estimates are roughly in line with data on safely and unsafely managed sanitation in Nairobi (42), sewer coverage (74) as well as emptying practices of onsite sanitation and use of shared sanitation facilities (75).

Table 3: Approximate quantitative distribution of population per sanitation service regime in Nairobi - present situation rounded to 10,000s (based on 2022 population raster data (41) and sanitation service regime allocation given in Figure 3)

	COPING	PUBLIC TOILET	ST/NC	CONTAINER	ST/SEWER	HT/NC	HT/RT	HT/SEWER	SUM
%	2	2	10	3	23	30	8	23	100
Total	70,000	70,000	450,000	150,000	1,050,000	1,370,000	350,000	1,050,000	4,560,000

Our estimates show that less than a third of the population in Nairobi is provided with a 'good' sanitation service from an individual and neighbourhood perspective (as constituted by the HT/RT or HT/SEWER regimes). We estimate that around 40% of the population relies on services that are problematic from the perspective of personal safety and convenience or public health protection in urban neighbourhoods (COPING, PUBLIC TOILET, ST/NC or HT/NC). We would also like to highlight that whilst we treat shared sanitation service regimes that effectively remove excreta from the neighbourhood (CONTAINER and ST/SEWER) as acceptable intermediate solutions, these systems do not always constitute good sanitation from an individual and public health perspective. We have already discussed the public health risks from sewage seepage or overflow. In addition, particularly for low-income and informal areas, there are reports of exceedingly high numbers of people sharing toilets (43, 76) and the regulator noted a trend for a rising number of people per sewer connection which raises concern in terms of the quality for the user (74). Getting reliable estimates of the number of container-based sanitation units under the Freshlife franchise is challenging. Still, in October 2023, a figure of 5,500 was provided, although, by Fresh Life's own admission, the numbers fluctuate monthly. Emptying of these units is done under the control of Sanergy, and hence, public health risks are minimised. Most units are shared between multiple households. The Sanergy system continues to be substantially subsidised by donor funding, and so far, attempts to agree with NCC on a publicly subsidised service contract have not been successful (30, 33, 65).

Climate change effects on failures and distributional inequity within Nairobi sanitation system

Having outlined the current sanitation situation in Nairobi and some of the general challenges and failures of the current sectoral regime, we will now analyse how climate change will likely affect the sanitation service situation in Nairobi.

We, hence, combined the HyCRISTAL scenarios outlined in the methods section (Table 1) with insights on climate change impacts on urban sanitation failures (12) to contextualise the effect climate change is likely to have on Nairobi's stressed sanitation infrastructure. Table 4 summarises relevant climate impacts along the sanitation service chain for each service regime, highlighting existing pressure.

Table 4: Relevance of climate change impacts along the sanitation chain (based on (12) under different future climate scenarios established by the HyCRISTAL

SERVICE REGIME	ACCESS / CONTAINMENT	EMPTYING / TRANSPORT	TREATMENT	DISPOSAL / ENDUSE
HT/SEWER	Alternative (potentially unsafe) sanitation options are used as coping mechanisms due to failing water-based toilets during periods of water restrictions<br>**F3**	Increased risk of pipe damage due to changed soil moisture and subsidence<br>**F1**<br>Increased inflow and infiltration into the sewer system (particularly into dilapidated pipes and through missing manhole covers)<br>Sewer blockages after and event because of sand, debris or solid waste entering sewers and pump stations<br>Electricity failure leading to failure of pumps<br>Damage to sewer mains and pumps<br>Overload of sewer system resulting in overflow to the drainage system<br>**F1,** F2, F3<br>Higher risk of blockages in the sewer and drainage system and discharge pipes<br>Higher risk of corrosion of sewers<br>Pipe and joint breakages through ground settlement after prolonged droughts<br>(+) Decreased risk of urban flooding<br>**F3**	Flooding and damage to WWTPs structure and equipment<br>Flooding of WWTP leading to temporary system failure and discharge of raw sewage<br>Electricity failure leading to failure of pumps and aeration<br>Road interruptions leading to disruption of site access for WWTP staff and supplies<br>Pollutant load exceeding biological treatment capacity of WWTP<br>Reduced nutrient removal capacity during high intensity rainfall<br>**F1,** F2, F3<br>Groundwater inflow and infiltration into wastewater pipes causes higher inflow into WWTPs and further stretches their insufficient treatment capacity<br>**F1**<br>Higher concentration of wastewater leading to less effective treatment<br>Corrosive influent damages equipment in treatment plants<br>Excess deposition due to low flow<br>**F3**<br>Moderate increases in temperature might increase efficiency of biological WWT<br>F1, F2, F3	Discharge of untreated/partially treated effluent due to overloading or bypassing of treatment<br>Contamination of receiving water bodies due to WWTP failure<br>**F1,** F2, F3<br>(+) Increased dilution of influent<br>**F1**<br>Less dilution in receiving waters<br>**F3**
ST/SEWER	Damage to superstructure making toilet unusable<br>Toilets become inundated/inaccessible (causing people to (temporarily) abandon toilet<br>**F1,** F2<br>Alternative (potentially unsafe) sanitation options are used as coping mechanisms due to failing water-based toilets during periods of water restrictions<br>**F3**			
HT/RT	Backflow of sewage from septic tanks<br>Damage to pits, septic tanks and absorption fields<br>Inundation of drain fields<br>**F1,** F2, F3<br>Floatation and damage of septic tanks due to high groundwater table<br>**F1**<br>Low moisture content of soil leading to erosion and damage of subsurface structures<br>**F3**	People 'drain' toilets during flood events leading to faecal contamination<br>Limited access for emptying services due to:<br>- Structural damage to pavements and other road elements such as bridges<br>- Road collapse or development of sinkholes due to destabilisation of soil caused by damaged sewer pipes Roads become inundated/inaccessible<br>- Decreased road capacity / increased congestion / increased travel time<br>**F1,** F2, F3<br>Limited access for emptying services due to heat damage to access roads<br>F1, F2, **F3**	Flooding and damage to FSTP<br>Road interruptions leading to disruption of site access for FSTP staff and supplies<br>Flooding and damage to wetland flora<br>**F1,** F2, F3<br>(+) Moderate increases in temperature might increase efficiency of FST in septic tank systems and FSTPs<br>F1, F2, F3	Higher groundwater/surface water pollutant risk caused by increased mobility of pollutants from septic tank drainfields<br>**F1**

SERVICE REGIME	ACCESS / CONTAINMENT	EMPTYING / TRANSPORT	TREATMENT	DISPOSAL / ENDUSE
ST/NC AND HT/NC	*For septic systems see above*<br>Damage to superstructure making toilet unusable<br>Toilets become inundated/inaccessible (causing people to (temporarily) abandon toilet<br>Damage/collapse of pits<br>Flooding of pits from below<br>**F1,** F2, F3<br>Low moisture content of soil leading to erosion and damage of subsurface structures<br>(+) Lower GW pollution risk from pit latrines<br>**F3**	People 'drain' toilets during flood events leading to faecal contamination<br>**F1,** F2, F3	(+) Moderate increases in temperature might increase efficiency of FST in septic tank systems and FSTPs<br>F1, F2, F3	Higher groundwater/surface water pollutant risk caused by increased mobility of pollutants from septic system drainfields<br>**F1**
CONTAINER (ST/RT)	Damage to superstructure making toilet unusable<br>Toilets become inundated/inaccessible (causing people to (temporarily) abandon toilet<br>**F1,** F2, F3	Limited access for emptying services due to:<br>- Structural damage to pavements and other road elements such as bridges<br>- Road collapse or development of sinkholes due to destabilisation of soil caused by damaged sewer pipes Roads become inundated/inaccessible<br>- Decreased road capacity / increased congestion / increased travel time<br>**F1,** F2, F3<br>Limited access for emptying services due to heat damage to access roads<br>F1, F2**, F3**	Flooding and damage to treatment facility<br>Road interruptions leading to disruption of site access for treatment facility staff and supplies<br>Flooding and damage to wetland flora<br>**F1,** F2, F3	
PUBLIC TOILET (PT)	Damage to superstructure making toilet unusable<br>Toilets or access roads become inundated/inaccessible (causing people to (temporarily) abandon toilet<br>**F1,** F2, F3		*Depending if connected to sewer or septic system - refer to sections above*	
COPING (NT/NC)	General unhygienic conditions during flooding increase risk of faecal contamination of surroundings from open defecation and other unsafe sanitation practices<br>**F1,** F2, F3		*N/A*	

Likely to be relevant for specific climate future scenario: F1 - Future 1: Much wetter, large increase in extreme rainfall and hotter; F2 - Future 2: Increase in extreme rainfall and hotter; F3 - Future 3: Much hotter and drier with more erratic rainy seasons (**bold:** likely to be highly releveant for specific climate future scenario)

(+) Positive climate impact

The synthesis shows that for all sanitation service regimes in Nairobi, flooding – especially flash floods – emerges as a critical concern under all three climate scenarios. In Nairobi, stormwater is managed through both natural and artificial drainage systems. Artificial stormwater drainage includes (where available) combined sewer pipes and roadside drains. Stormwater drains are frequently blocked by solid waste, leaves and debris, and the lack of maintenance of Nairobi's drainage system is a longstanding problem (77, 78). With substantial technical data on the existing stormwater drainage system being lost, there is no comprehensive urban stormwater management plan so far (79, 80). In addition, some pit latrines – or other toilets relying on subsurface containment – are illegally drained during heavy rainfalls and floods, contributing to neighbourhood-wide faecal contamination. Exhauster trucks and other emptying services get disrupted when roads are flooded or damaged during extreme rainfall (61). People who do not have toilets within their accommodation might have temporarily limited or no access to their toilets during floods or lose access completely if toilets get damaged. Untreated or partially treated sewage (from sewer overflows or overloaded treatment facilities) contributes to environmental and particularly river pollution (81).

Urban flood modelling is complex and often restricted by the coarse granularity of global models, which neither sufficiently capture susceptibility factors nor are these models suitable to assess real vulnerability or adaptive capacity of the exposed urban areas (82-84). We compared the 2010 GIS land use classifications by Ledant (29) with a current basemap showing further urban densification along flood- and landslide-prone riverbanks. Major housing developments (formal and informal) have occurred in many formerly undeveloped or agricultural areas in Nairobi. It is expected that most of the population increase will be in the low and middle-income areas in the eastern parts of the city (37, 85), such as the Kasarani sub-county which are highly susceptible to frequent flooding even under current climatic conditions and where rapid property development will also substantially increase the proportion of paved surface area (86).

More erratic rainfall patterns leading to prolonged duration of dry periods or drought will particularly stress the effective operation of piped-based systems (HT/SEWER and ST/SEWER) as they are likely to contribute to higher risk of blockages and corrosion in sewers and treatment facilities and lower dilution capacity of wastewater receiving waters amongst others (12). NCWSC is currently unable to

meet the population's water demand (87). As demand has outstripped water supply in Nairobi for decades, Ledant (29) attests that "Nairobi is in a situation of structural water shortage likely to worsen until further expansion" (29, p.338). While substantial investments have been made into Nairobi's water supply system (87), climate change is already an additional stressor for the water supply and sanitation systems. In the last decade, Kenya has suffered from extensive drought, which has affected Nairobi's water supply. The supply rationing led to a drop in NCWSC's revenue, which contributed to reduced budget allocation for sewer expansions in the informal settlements (87). Whilst middle and higher-income customers often have sufficient domestic water storage tanks to cope with interrupted water supply, low-income residents often do not have the same backup and therefore, sewer systems in low-income areas (ST/SEWER) will be most severely affected by increased water shortage under climate change (88). Simplified sewer systems (SSSs) that require less water and are cheaper to implement have been piloted in parts of Mukuru informal settlements. SSSs are not a recent technology and are widely used globally, mainly in Latin America. The existence of trunk sewers in many low-income areas of Nairobi offers favourable implementation opportunities. However, a robust evaluation of the scalability and sustainability of these systems in Nairobi, particularly concerning institutional willingness and financial allocations, needs further research (89).

Gaps between policy and implementation

In the following sections, we present the findings of our review of legal, policy, and investment documents, focusing on how these documents recognise and address equity concerns. However, we would like to embed our analysis in a more general debate of gaps between policy and implementation – which is not a Kenya or sanitation-specific problem but a global phenomenon observable at all levels of state or organisational governance.

In urban planning and, specifically, climate resilience, the key to understanding these gaps is the concept of 'fantasy plans' (90), describing policy aspirations that are ambitious and possibly idealistic but disconnected from practical and achievable implementation strategies. This often leads to formulating policies that are more symbolic and performative than substantive, creating an illusion of progress without tangible results (90, 91). In the context of basic service delivery, on paper, such policies

appear to recognise equity challenges; however, by adopting policies that are not grounded in reality, governments risk perpetuating inequities and failing to change the distributional pattern of sanitation services genuinely.

Linking climate change and sanitation in policy and planning –mainstreaming or out-of-sight?

Our review showed that within the current national and county-specific legal and policy framework, climate adaptation is not adequately reflected – and budgeted for – in sanitation plans, and sanitation is barely considered in climate change adaptation law and policies.

The National Climate Change Act (92) requires that County Governments mainstream climate change actions and interventions in their County Integrated Development Plans (CIDPs) but does not reference the water and sanitation sector. NCC's current CIDP makes no concrete links between climate adaptation and sanitation. Hence, no climate resilience, adaptation, or mitigation strategies exist for the sanitation sector within the current county planning (93). On the national level, there are no specific sanitation-related commitments to adaptation or mitigation in Kenya's Nationally Determined Contributions (NDCs) (94). The National Adaptation Plan (NAP) (95) offers only vague actions for the water and sanitation sector (S1 Table). It links long-term actions to the National Water Master Plan (96), which focuses on water resource management. As such, it lacks a specific focus on climate adaptation within the sanitation sector in the Athi Catchment.

Within the water and sanitation sector, specific legal and policy framework (S2 Table) climate change adaptation is primarily considered (if at all) in the context of water resources/water security and (less frequently) urban drainage or flood resilience. Two of the most recent sector documents – the National Water and Sanitation Service Strategy (2020-2025) (97) and the National Sanitation Management Policy (the reviewed document is a report documenting the process of developing the policy available as the draft policy that is currently debated could not be obtained) (98) – show an increased recognition of the potential impacts of climate change on the sanitation sector.

The supplemental information (S1-S3 Table) lists all reviewed legal, policy and planning documents and summarises their respective consideration of climate change adaptation in the context of urban sanitation.

Is there any money for climate adaptation of sanitation?

Evaluating budget allocations offers insight into governments' commitment to equitable and long-term provision of basic services beyond performative pledges. A detailed assessment of the climate finance landscape in Kenya (99) revealed a primary government focus on investments in mitigation efforts, especially in the renewable energy sector. This is despite the NAP (95) and the NDCs (94) emphasising adaptation strategies. Although investment in the water and wastewater management sector was the most substantial in terms of adaptation in 2017/18, it still falls short of the requirements for water sector adaptation outlined in the NAP (95). Importantly, the climate finance landscape assessment has no further subdivision between the water and wastewater sub-sectors. Given the current policy focus (see above), it is probable that most investments were directed towards initiatives to alleviate water scarcity.

On the county level, the apparent disconnect between sanitation and climate change policy, as discussed earlier, aligns with the absence of explicit links between climate change and urban sanitation in Nairobi City County's (NCC) urban planning and budgeting frameworks (70, 93, 100). Under the institutional framework of the Kenyan water sector, the Athi Water Works Development Agency (AWWDA) is responsible for developing sewerage infrastructure, whilst Nairobi City Water and Sewerage Company (NCWSC) manages and maintains water and sewerage services in Nairobi City County (101). Both entities display only limited and general references to climate change adaptation in their most recent investment plans (87, 102).

Kenya's water sector heavily depends on donors, with approximately 70% of the annual capital investment coming from development partners (103). In addition, Kenya expects that implementing their NDCs will require international partners to sustain at least 87% of the costs by 2030, a currently unmet target (99).

The reviewed investments by the development partners (S3Table) all incorporate considerations for climate resilience in infrastructure design, primarily aimed at mitigating flood risks through enhanced

drainage and sewer systems. However, none of the projects explicitly mention climate change adaptation in their outcome indicators (71, 104-107). The Green Climate Fund's Infrastructure Climate Resilient Fund does not directly finance the sanitation sector. While its proposed investments in road and bridge rehabilitation could indirectly benefit sanitation services dependent on road transport, specific details about the locations of these investments remain unclear (108).

Adaptive sanitation services for whom? Equity considerations in the current sanitation and climate change adaptation planning framework

We now turn towards a deeper examination of the equity considerations within the policy and planning framework for sanitation climate adaptation in Nairobi. Our initial spatial and quantitative analysis highlighted existing distributional inequities within Nairobi's present sanitation service regime configuration. As previously noted, climate change will put an uneven burden on urban populations and is likely to worsen the challenges of low-income and marginalised groups and exacerbate existing inequities (109, 110).

We have found a broad disjoint between climate adaptation and sanitation service planning in the current policy framework and budget allocations. Although climate change adaptation is not explicitly integrated into sanitation policies, and vice versa, addressing urban vulnerabilities in both areas could still help prevent climate change from intensifying sanitation inequities. Thus, in the following, we explore if and how the three dimensions of sanitation adaptation equity (Table 2) are incorporated within the policy, planning and investment framework.

Our findings suggest that, despite formal recognition of sanitation service inequalities and the needs of marginalised groups within various policies, this awareness seldom translates into actions prioritising and effectively enhancing distributive equity in sanitation service across the city. This echoes Weinstein et. al's (90) assessment of flood resilience planning in Mumbai: "Yet while the discourse has shifted to include some language [...], the specific developments it proposes do not suggest an altered development context" (90, p.274) Moreover, we recognise that assessing procedural equity is

challenging when solely analysing legal and policy documents. We draw on existing scholarly work to explore the misalignments within Nairobi's divided sanitation system. This analysis highlights how these misalignments further entrench sanitation inequities within the context of broader structural and procedural inequities

Recognition. Over the last two decades, Kenya has made some progress in recognising the needs of low-income populations within its water and sanitation governance framework. The Water Sector Trust Fund was established in 2002 to increase investment in water and sanitation for low-income and marginalised population groups. The National Water Services Strategy (2007-2015) and the Pro-Poor Implementation Plan for Water Supply and Sanitation, adopted in 2007 (111), signalled a commitment to a pro-poor approach in water and sanitation service delivery. The Kenyan constitution, amended in 2010, includes the human right to water and sanitation (31, 112). In 2018, the national water sector regulator WASREB introduced a 'Pro-poor' Service Assessment in the annual utility reporting (113). However, the respective indicator is not part of the core performance ranking metrics. Most sanitation investments in low-income areas heavily rely on external funding (31). Acknowledging the persisting funding gap for sanitation development, there have been longstanding discussions in Kenya about introducing a levy to fund the sewer system expansion and investment in non-sewered sanitation (97, 101). In 2019, WASREB has published guidelines for structuring such a levy (114). However, no Water Service Provider (WSP) has implemented this levy, and its execution appears unlikely due to the recent backlash against the housing levy and the ongoing global cost of living crisis (115).

Similarly, the climate change-related policy framework generally recognises the vulnerability of poor urban neighbourhoods (e.g., NCCRS, 2010 (116), NCCAP, 2018 (117)) but pledges to fair distribution, such as the NPCF's (2016) commitment to 'equitable benefits sharing' remain vague and unspecific.

Distributional. Within the sanitation policy and planning framework of Nairobi, the identified references to the concept of distributional equity are primarily related to the evaluation criterion: '*Which sanitation service configurations are represented in the current or planned adaptation* measures' (Table 2). Notably, a considerable portion of the legal and policy framework, including the National Water Master Plan (NWMP) (96), the Water Act (101), the CIDP (93) and the Nairobi Integrated Urban Development Plan (NIUPLAN) (70) predominantly overlooks non-sewered sanitation service regimes.

This omission effectively excludes populations for whom a connection to the sewer system is not attainable in the near future

The prevailing sanitation sector investment strategy, adhering to the paradigm of the *'networked city'* (118), concentrates on sewer network expansion aiming to increase sewerage coverage in Nairobi from 50% to 80% by 2030 (93) and on rehabilitating the existing sewer network that has suffered from insufficient maintenance for decades. Despite the ongoing and planned investments, such a substantial increase in coverage within this period appears unrealistic. Whilst the Kenyan Government has revised its national sewerage coverage target from a fantastic 80% by 2030 to a more balanced (but likely still over-ambitious) goal of 40% sewerage and 60% safe onsite sanitation (74), the NCC and NCWSC have yet to adjust their targets. Due to rapid population growth, the development of sewerage infrastructure has not kept pace with this rapid population increase and therefore, the sewerage coverage rate has remained stagnant at 50% since 2015 (45, 113). Importantly, coverage also only describes the existence of a sewer network and should not be equated with people being connected to (i.e., accessing) sewered-based sanitation (119).

Plans and investments dedicated to improving the sanitation situation for those who will not be connected to the sewer network in the near future, especially in terms of climate resilience, are markedly limited. The water service provider and the county have shown limited consideration for heterogeneous sanitation service regime configuration. The NWMP (96) typifies the endemic vagueness characterising non-sewered sanitation planning in Nairobi. The NWMP's sanitation annex for the Athi catchment area, which includes Nairobi, vaguely declares:

> "Outside the sewerage service area, the improved on-site treatment facilities will be available for the remaining 4.28 million residents in 2030. […] unimproved facilities will be improved with new housing. Development of on-site sanitation facilities is planned for the ten counties in ACA." (96)

From the information in the NWMP however, it is not clear who will be responsible for planning, implementing, and paying for these improved on-site sanitation facilities. Although more recently, Kenya has (at least on paper) committed to Countywide Inclusive Sanitation (COWIS) planning (114,

120), and a feasibility study for the Nairobi Inclusive Sanitation Improvement Project (107) is underway, a concrete vision for managing and funding non-sewered sanitation programs is still absent. Almost all public investments in non-sewered sanitation in Kenya depend on external funding both for capital and operational expenditures (30, 114), illustrating their similarity to sewered sanitation systems, which rely on continuous public or other funding for sustained operations and maintenance. Thus, without government commitments to take on their operations, such systems remain unsustainable (7, 121)

Unlike some other Kenyan water utilities – such as the utilities in Kisumu, Nakuru or Malindi (Peletz et al., 2020; Grisaffi et al., 2022) – NCWSC has shown little enthusiasm for engaging in non-sewered sanitation service provision (30). In their water and sanitation planning, NCC merely incorporates the sewerage-focused plans from the NCWSC and commits to constructing some public toilets (93), without showing any meaningful engagement with any non-sewered sanitation service regimes. Service providers such as NCWSC are often reluctant to engage in non-sewered sanitation or to work in low-income areas, often citing the lack of profitability of such endeavours, despite the lack of evidence that any sanitation interventions, including sewerage, can or indeed should be fully self-financing (121). Various scholars have noted that the prevailing institutional incentives and regulatory frameworks prioritise utilities' operational and financial efficiency over the constitutional right to water and sanitation (29, 122). Kenya's urban water sector is undergoing a commercialisation process, and – despite rhetorical commitment to COWIS – service providers are encouraged to attract private sector investment and formulate 'bankable' projects not only by government policy but also by development partners (122, 123). In the absence of structured public finance for adequate sanitation service provision outside the sewered sanitation service regimes, the strong focus on cost-recovery conflicts with the public health benefits of adequate sanitation and has been identified as a major hindrance in promoting citywide sanitation (7).

Procedural: Regarding procedural equity, our analysis exposed predominately ambiguous rhetoric about 'inclusive planning' in policy formulation processes (e.g., 124) or equally unspecific commitments to 'extensive consultations' that should occur in further planning (e.g., 94). The Nairobi City County

(NCC) admits to the low public inclusivity in the previous County Integrated Development Plan (CIDP) (2018-2022) and broader NCC activities yet fails to outline specific strategies for enhancing public engagement in the implementation of the current CIDP (93). Scholars have characterised Kenya's urban planning governance as 'exclusionary' and 'technocratic' (125, 126), noting that Nairobi's urban development, including water and sanitation provision, remains influenced by colonial legacies of segregation and exclusion (36, 126). Despite growing recognition of non-sewered sanitation (see above), there is still no clear institutional home, coherent governance framework and political will to deal with the planning and management of non-sewered sanitation, particularly in informal settlements (31, 33). Additionally, strong vested interests persist in maintaining unregulated informal sanitation services controlled by well-connected and exploitative 'cartels' in the low-income areas of Nairobi (61, 127). Sanitation planning in Nairobi is not well linked to other relevant urban sectors such as housing and land-use planning, transport and energy planning (44). Even the interdependencies between sanitation with the most closely related urban sector, water supply, are not adequately considered. Water-based sewerage networks are still the only official sanitation strategy of NCWSC (87). However, water demand in Nairobi has long outstripped supply. As a result, water rationing has become part of the official supply strategy of NCWSC and insufficient water pressure is an increasing challenge for quickly developed new high-rises for the middle class (66, 128).

On paper the Mukuru Special Planning Area (SPA) presents a notable exception. The informal settlement upgrading project initiated in 2017 officially embraces all of the three aspects of sanitation adaptation equity, particularly in its inclusive processes. The designation of Mukuru as an SPA led to the suspension of conventional planning regulations, acknowledging their inadequacy for the settlement's unique challenges and enabling more context-specific solutions (125). While not presented as a best-practice example, the Mukuru SPA's efforts in facilitating resident co-design of climate change adaptation measures, including improvements in flood resilience, drainage, and sanitation systems (129), are commendable within an overall exclusionary urban governance framework (125). The NCWSC piloted a limited number of simplified sewer connections in Mukuru, which are more suitable for pour-flush toilets, intermittent water supply, and drought resilience (89). Separately, Fresh Life provides container-based sanitation systems in the area.

However, the costs of the connections to the simplified network have been criticised by residents and local leaders (64) and many Mukuru residents still face inadequate sanitation, with high numbers sharing toilets and people reverting to buckets or other forms of unsafe sanitation due to the long distances to toilets, particularly at night (129, 130). The implementation of the co-designed SPA proposals has been delayed and is still ongoing, precluding a comprehensive evaluation. Generally, the value of the co-design process has been acknowledged by various researchers (125, 129, 131), although NCC engagement in facilitating the participation of residents has been criticised as insufficient (131) and residents of Mukuru still feel that the government does not adequately engage with improving their sanitation situation, prioritising instead the more affluent areas in the city (64). One of the authors who visited Mukuru SPA in February 2024 could not identify any evidence to suggest systemic or transformative engagement of NCC in Mukuru and summarised the limited reach of external partner-initiated sanitation investments as "[it] is like sprinkling a bit of glitter on a rubbish dump."

Estimate of future conditions

The evidence from our projections of the future trajectory predominantly suggests a business-as-usual approach to sanitation service provision in Nairobi. This implies that substantial reconfiguration or realignment of sanitation service regimes is unlikely; therefore, the sector is expected to remain fragmented, with marked inequities in access to and quality of sanitation services.

Despite ongoing investments in conventional sewerage, which will increase the number of individuals with sewer access; primarily by connecting shared toilets to sewers (ST/SEWER), these efforts are projected to be substantially offset by rapid population growth in Nairobi. Assuming a 2030 population of 6 million (77), maintaining the current 50% sewerage coverage would require connecting approximately 700,000 people, or about 90,000 people annually from 2022 to 2030. To achieve the NCC target of 80% sewerage coverage by 2030, the number increases to 280,000 people annually.

Our analysis found no substantial evidence suggesting that non-sewered sanitation systems will be more effectively organised by 2030. Therefore, our projected spatial distribution for Nairobi in 2030 under a business-as-usual scenario incorporates the planned sewer extensions outlined in the Nairobi

Integrated Urban Development Master Plan (NIUPLAN) (70). We expect minimal changes to the overall spatial distribution of sanitation service regimes beyond these extensions (Figure 4).

Figure 4: Approximate spatial distribution of dominant sanitation service regimes in Nairobi – projection for 2030 (based on information about sewer development from Nairobi Master plan and planning framework detailed in S2 Table): COPING (NC/NT) = People don't have access to toilets us coping mechanisms; PUBLIC TOILET = People rely on public toilets as main form of sanitation; ST/NC = Shared toilets that are NOT adequately managed; CONTAINER (ST/RT) = Shared toilets that are regularly emptied; ST/SEWER = Shared toilets connected to the sewer system; HT/NC = Household that are not adequately managed; HT/RT = Household toilets that regularly emptied; HT/SEWER = Household toilets connected to the sewer system

The expected modest shifts in the spatial distribution of service regimes indicate that the proportional distribution of individuals using these services will largely depend on the geographic pattern of population growth. At the time of writing, the Kenya National Bureau of Statistics (KNBS) had not released official population projections based on the 2019 census data. Our total population figure of 6.1 million is consistent with the estimate of 6 million people that is cited by the UN-Habitat (132) and The World Bank (133, 134). Detailed statistical population forecasting was beyond the scope of our study. Still, our spatial analysis and policy review support the plausibility that by 2030, more Nairobi residents will rely on sanitation services that neither provide a good service from the individual perspective nor protect public health within the direct proximity nor are resilient to climate change impacts, particularly urban flooding (Table 5).

Table 5: Comparision of the estimated distribution of population per sanitation service regime in Nairobi in 2022 (baseline) and 2030 rounded to 10,000s

	Baseline 2022 (see Table 2)		*Forecast for 2030*		*Delta*	
	Total	Proportion in %	Total	Proportion in %	Total	
COPING	70,000	2	120,000	2	+50,000	-
PUBLIC TOILET	70,000	2	120,000	2	+50,000	-
ST/NC	450,000	10	730,000	12	+280,000	+2
CONTAINER	150,000	3	230,000	4	+80,000	+1
ST/SEWER	1,050,000	23	2,100,000	34	+1,050,000	+11
HT/NC	1,370,000	30	1,120,000	18	-210,000	-12
HT/RT	350,000	8	370,000	6	20,000	-2
HT/SEWER	1,050,000	23	1,340,000	22	+290,000	-1
Total	4,560,000	100	6,130,000	100	+1,010,000	

Our projections for 2030 sewerage coverage fall considerably below NCWSC's target figures. However, given the longstanding stagnation in sewer coverage rates and the challenge to meet existing water demand in Nairobi as well as documented challenges of water utilities in Kenya and elsewhere incentivising connections to existing sewers (119, 135), we believe our projection of approximately 1.3 million additional people covered by sewerage by 2030 is optimistic. In fact, our model, based on proximity to sewer lines and income levels, may overestimate sewer connections (Figure 1).

We also acknowledge that our estimated prospects for expanding container-based sanitation services (CONTAINER regime) fall short of business expansion plans or the involved providers. We found no evidence of sustained funding that could drive a more significant expansion of road-based sanitation services within the current governance framework. The expansion of such services depends on household investments and external funding, and long-term sustainability will require public finance, which is not evident in current planning (33, 64, 65). Currently, public finance for sanitation in Nairobi predominantly subsidises sewerage.

The impacts of climate change on all urban sanitation service regimes (Table 4) increasingly threaten service sustainability. Without targeted measures to enhance flood resilience, parts of Nairobi's population face escalating risks of sanitation system damage, malfunction, and temporary to permanent loss of access to adequate sanitation systems. Previous research shows that poor households often struggle to adapt their sanitation systems, reverting to unsafe practices in response to increased flooding (136-139). Consequently, it is plausible to expect an increase in low-income residents relying on coping strategies or public toilets due to more frequent flooding. We chose not to quantitatively incorporate this factor to avoid suggesting false precision amidst cascading uncertainties. On the other hand, it is worth noting that expanding sewer systems and drainage in a coordinated manner (either through separated systems or well-designed combined sewers) could reduce urban flooding, provided that both systems are properly maintained and remain free from debris and solid waste blockages – a current challenge in Nairobi.

Limitations

We acknowledge that our estimates on sanitation service configuration across Nairobi have considerable limitations as they are based on a coarse allocation of dominant sanitation service regimes for sub-areas. Consequently, both our spatial distribution and quantitative estimates should not be interpreted as precise.

We further recognise several limitations in our forecast of the sanitation regime configuration for 2030. Population projections are hugely complex. Elaborate statistical population forecasting was beyond the scope and intent of this study. The relatively short time series available for our forecast limits the reliability of the projections. In addition, our spatial model of the sanitation configurations consists of relatively small subareas, which further challenges the reliability of the associated raster data projections. As stated earlier, we provide a detailed description of our forecasting method and critical assessment of the limitations of our projection in the supplemental information (SI 4).

However, we remain confident that imprecisions in our quantifications do not substantially challenge the validity of our general findings, which are backed by our comprehensive analysis and review of the sanitation planning framework as well as the wider literature.

Conclusion

In this paper, we have explored the rhetoric and realities of addressing sanitation inequities in Nairobi, particularly in the context of climate change adaptation. Nairobi's splintered sanitation sector is increasingly at risk due to the impacts of climate change. This vulnerability threatens to deepen the precarious and deeply inequitable sanitation service situation.

Our comprehensive review of Nairobi's legal, policy, and investment documents uncovers a critical gap between climate change and sanitation policy and planning. The evidence starkly highlights that the current policy and service provision are failing to deliver high-quality, equitable, and resilient sanitation services. Despite high-level government commitments (mainly on national government level) to ambitious targets concerning these aspects, a significant disconnect exists between the official pledges and the actual implementation on the ground. This gap is not only evident but is set to widen,

deteriorating the sanitation experience for Nairobi's citizens as urbanisation, population growth and climate change impacts accelerate.

This analysis brings to light the concept of 'extraversion,' suggesting that the current political economy may foster a culture where development partners, government actors, and (international) non-governmental organizations collectively engage in practices that mainly improve the wording in well-intentioned policy and framework but avoid addressing the more challenging aspects of sanitation service provision and their societal root causes (90, 91, 140).

Despite acknowledging the necessity for service equity in Kenya's 'pro-poor' water and sanitation policy framework, there is a lack of effective measures to shift away from a sewer-centric sanitation governance model. Instead, there is a glaring absence of a comprehensive citywide sanitation delivery framework, along with insufficient investment and operational subsidies for non-sewered sanitation service regimes. Consequently, sanitation planning in Nairobi seems destined to persist with a *'business-as-usual'* approach, prioritising sewerage development and neglecting the fragmented reality of its sanitation services as well as continuous (and often unplanned) urbanisation and increasing climate impacts on basic urban services.

Synthesising all findings from our analysis leads us to conclude that due to the inadequate integration of climate change and equity considerations in Nairobi's sanitation planning, it is highly plausible that by 2030, a substantial portion of residents will continue to depend on poor sanitation services. These services will likely fall short of providing good individual-level service quality and for safeguarding public health, particularly in the face of escalating climate threats.

The gaps exposed by our analysis are not limited to the local case of Nairobi. Splintered sanitation service regime configurations can be found in many major urban centres across Sub-Saharan Africa (28) and with modifications in low- and lower-middle income countries globally. Equally, 'fantasy planning' and extraversion are global phenomena. The congratulatory exchanges among government and development actors at policy forums and climate summits, while promoting policy advancements, do not address the core challenges in service provision.

Our analysis, therefore, underscores the urgent need for a fundamental shift in approach. There must be an honest and robust discussion about providing high-quality, resilient services at scale beyond sewered areas, necessitating substantial government funding and support.

Acknowlegments

The authors would like to thank Lais Dos Santos and Virginia Roaf for their constructive feedback on earlier versions of this manuscript. Our gratitude also goes to Ana Casas Garcia, who offered advice on population forecasting from a statistical viewpoint, and to Bart Hill for his guidance on the application of ArcGIS tools in population forecasting. Our thanks extend to Martin Ledant and Mutono Nyamai for their generosity in sharing shapefiles from their previous research on water inequalities in Nairobi. Additionally, we are deeply thankful for conversations with Anne Aol and Elizabeth Wambui, which offered invaluable insights into sanitation issues in Nairobi.

References

1. WHO and UNICEF. Progress on household drinking water, sanitation and hygiene 2000–2022: special focus on gender. New York: United Nations Children's Fund (UNICEF) and World Health Organization (WHO); 2023.

2. Brown J, Acey CS, Anthonj C, Barrington DJ, Beal CD, Capone D, et al. The effects of racism, social exclusion, and discrimination on achieving universal safe water and sanitation in high-income countries. The Lancet Global Health. 2023;11(4):e606-e14.

3. WHO, UNICEF. Progress on household drinking water, sanitation and hygiene 2000-2017. Special focus on inequalities. New York: United Nations Children's Fund (UNICEF) and World Health Organization; 2019.

4. Del Duca L, Dickin S, Macura B, Soto Trujillo A. SEI Perspectives [Internet]: Stockholm Environmental Institute. 2020. [cited 2023]. Available from: https://www.sei.org/perspectives/covid-19-and-wash/.

5. Howard G, Bartram J, Brocklehurst C, Colford JM, Costa F, Cunliffe D, et al. COVID-19: urgent actions, critical reflections and future relevance of 'WaSH': lessons for the current and future pandemics. Journal of Water and Health. 2020;18(5):613-30.

6. Nath P, Gosling L. Washmatters [Internet]: WaterAid. 2020. [cited 2023]. Available from: https://washmatters.wateraid.org/blog/putting-equality-inclusion-and-rights-at-centre-of-covid-19-water-sanitation-and-hygiene-response.

7. Gambrill M, Gilsdorf RJ, Kotwal N. Citywide Inclusive Sanitation—Business as Unusual: Shifting the Paradigm by Shifting Minds. Frontiers in Environmental Science. 2020;7.

8. Hutchings P, Johns M, Jornet D, Scott C, Van Den Bossche Z. A systematic assessment of the pro-poor reach of development bank investments in urban sanitation. Journal of Water, Sanitation and Hygiene for Development. 2018;8(3):402-14.

9. Andres LA, Thibert M, Lombana Cordoba C, Danilenko AV, Joseph G, Borja-Vega C. Doing More with Less : Smarter Subsidies for Water Supply and Sanitation. Washington, DC: World Bank; 2019.

10. Mara D, Evans B. The sanitation and hygiene targets of the sustainable development goals: scope and challenges. Journal of Water, Sanitation and Hygiene for Development. 2018;8(1):1-16.

11. IPCC. Climate Change 2022: Impacts, Adaptation and Vulnerability. Contribution of Working Group II to the Sixth Assessment Report of the Intergovernmental Panel on Climate Change. Cambridge, UK and New York, NY, USA; 2022.

12. Hyde-Smith L, Zhan Z, Roelich K, Mdee A, Evans B. Climate Change Impacts on Urban Sanitation: A Systematic Review and Failure Mode Analysis. Environmental Science & Technology. 2022;56(9):5306-21.

13. Mikhael G, Hyde-Smith L, Twyman B, Sánchez Trancón D, Jabagi E, Bamford E. Climate Resilient Urban Sanitation: Accelerating the convergence of sanitation and climate action. Eschborn: Deutsche Gesellschaft für Internationale Zusammenarbeit (GIZ) and Resilient Cities Network; 2021.

14. Ensuring access to climate-resilient sanitation services for 3.6 billion people by 2030: A call to action for acceleration [press release]. UNICEF, the Global Green Growth Institute, the University of Technology Sydney, the Bill and Melinda Gates Foundation, UN-Habitat, the World Health Organization, the Asian Development Bank, the African Development Bank, Resilient Cities Network, WaterAid and SNV2023.

15. Dickin S, Bayoumi M, Giné R, Andersson K, Jiménez A. Sustainable sanitation and gaps in global climate policy and financing. npj Clean Water. 2020;3(1).

16. Hyde-Smith L, Zhan Z, Roelich K, Mdee A, Evans B. Blinded by the 'green-halo'? Equity in funding climate adaptation of urban sanitation. forthcoming.

17. Li J, Li X, Liu H, Gao L, Wang W, Wang Z, et al. Climate change impacts on wastewater infrastructure: A systematic review and typological adaptation strategy. Water Research. 2023;242.

18. Araos M, Jagannathan K, Shukla R, Ajibade I, Coughlan De Perez E, Davis K, et al. Equity in human adaptation-related responses: A systematic global review. One Earth. 2021;4(10):1454-67.

19. Schlosberg D, Collins LB. From environmental to climate justice: climate change and the discourse of environmental justice. WIREs Climate Change. 2014;5(3):359-74.

20. Schlosberg D. Theorising environmental justice: the expanding sphere of a discourse. Environmental Politics. 2013;22(1):37-55.

21. McCauley D, Heffron R, Stephan H, Jenkins K. Advancing Energy Justice: The triumvirate of tenets. International Energy Law Review. 2013;32:107-10.

22. Wood N, Roelich K. Substantiating Energy Justice: Creating a Space to Understand Energy Dilemmas. Sustainability. 2020;12(5):1917.

23. Schlosberg D, Collins LB, Niemeyer S. Adaptation policy and community discourse: risk, vulnerability, and just transformation. Environmental Politics. 2017;26(3):413-37.

24. Hall S, Roelich KE, Davis ME, Holstenkamp L. Finance and justice in low-carbon energy transitions. Applied Energy. 2018;222:772-80.

25. Hughes S, Hoffmann M. Just urban transitions: Toward a research agenda. Wiley Interdisciplinary Reviews: Climate Change. 2020;11(3).

26. Mullen C. Fairness in transitions to low-carbon mobility. One Earth. 2021;4(2):168-71.

27. Mullen C, Marsden G. Mobility justice in low carbon energy transitions. Energy Research & Social Science. 2016;18:109-17.

28. Van Welie MJ, Cherunya PC, Truffer B, Murphy JT. Analysing transition pathways in developing cities: The case of Nairobi's splintered sanitation regime. Technological Forecasting and Social Change. 2018;137:259-71.

29. Ledant M. Water in Nairobi: Unveiling inequalities and its causes. Les Cahiers d'Outre-Mer. 2013;263.

30. Zaqout M. The Political Economy of Sanitation An Exploratory Study of Stakeholder Incentives in Delivering Sanitation [PhD Thesis]. Leeds, UK: University of Leeds; 2023.

31. Mansour G, Oyaya C, Owor M. Situational analysis of the urban sanitation sector in Kenya. Nairobi: Water and Sanitation for the Urban Poor (WSUP); 2017.

32. Mdee A, Ofori A, Anciano F, Dube M, Kramer S, Lopez H, et al. The political economy of the container based sanitation off-grid 'solution': a sectoral and service regime analysis from Cape Town, Capatien, Lima, & Nairobi. forthcoming.

33. Mallory A, Mdee A, Agol D, Hyde-Smith L, Kiogora D, Riungu J, et al. The potential for scaling up container-based sanitation in informal settlements in Kenya. Journal of International Development. 2022.

34. Goodfellow T. Finance, infrastructure and urban capital: the political economy of African 'gap-filling'. Review of African Political Economy. 2020;47(164):256-74.

35. Iddi S, Akeyo D, Bagayoko M, Kiwuwa-Muyingo S, Chikozho C, Kadengye DT. Determinants of transitions in water and sanitation services in two urban slums of Nairobi: A multi-state modeling approach. Global Epidemiology. 2021;3:100050.

36. Nilsson D, Nyanchaga EN. Pipes and politics: a century of change and continuity in Kenyan urban water supply. The Journal of Modern African Studies. 2008;46(1):133-58.

37. Mutono N, Wright J, Mutembei H, Thumbi SM. Spatio-temporal patterns of domestic water distribution, consumption and sufficiency: Neighbourhood inequalities in Nairobi, Kenya. Habitat International. 2022;119:102476.

38. Luwe K, Sindall RC, Garcia-Becerra FY, Chinyama A, Lohiya H, Hope C, et al. Perceptions of Inclusivity and Sustainability in Urban Sanitation in Global South Cities. Environmental Health Insights. 2022;16.

39. Ledant M. Access to Water in Nairobi: mapping inequalities beyond statistics. In: UN-Habitat Ga, editor. 2013.

40. Google, cartographer Satellite imaginary of Nairobi2023.

41. Gadiaga AN, Abbott TJ, Chamberlain H, Lazar AN, Darin E, Tatem AJ. Census disaggregated gridded population estimates for Kenya (2022), version 2.0. In: Southampton. Uo, editor. 2023.

42. Sanergy, African Population and Health Research Center, Nairobi Water and Sewerage Company, Nairobi County Government. Mapping the urban sanitation challenge: The Nairobi SFD. Stockholm World Water Week; Stockholm2018.

43. Winter SC, Dreibelbis R, Dzombo MN, Barchi F. A mixed-methods study of women's sanitation utilization in informal settlements in Kenya. PLOS ONE. 2019;14(3):e0214114.

44. Sverdlik A. Nairobi: City scoping study. Manchester, UK: African Cities Research Consortium; 2021.

45. WASREB. IMPACT 15. A Performance Report of Kenya's Water Services Sector - 2021/22. Nairobi, Kenya: Water Services Regulatory Board; 2023.

46. Fresh Life. Fresh Life's own service estimates as of November 2023 provided by email to author 2023.

47. Bornemann FJ, Rowell DP, Evans B, Lapworth DJ, Lwiza K, Macdonald DMJ, et al. Future changes and uncertainty in decision-relevant measures of East African climate. Climatic Change. 2019;156(3):365-84.

48. FCFA. HyCRISTAL: Integrating Hydro-climate science into policy decisions for climate-resilient infrastructure and livelihoods in East Africa: Future Climate for Africa (FCFA); 2021 [Available from: https://futureclimateafrica.org/project/hycristal/.

49. Fathom. Fathom Global Flood Map Specification. Fathom; 2022.

50. Fathom. Fathom Global Flood Map. Methods: In brief. Fathom; 2022.

51. Bernhofen MV, Trigg MA, Sleigh PA, Sampson CC, Smith AM. Global flood exposure from different sized rivers. Natural Hazards and Earth System Sciences. 2021;21(9):2829-47.

52. Atteridge A, Savvidou G, Sadowski S, Gortana F, Meintrup L, Dzebo A. Aid Atlas: Stockholm Environment Institute (SEI); 2019 [Available from: https://aid-atlas.org/.

53. NCC. Integrated Urban Development Master Plan for the City of Nairobi. Nairobi, Kenya: Nairobi City County; 2014.

54. WorldPop. WorldPop Hub: Population Counts Southampton, UK: University of Southampton; 2024 [Available from: https://hub.worldpop.org/.

55. Li S, Liu J, Butler K. ArcGIS Blog [Internet]: ESRI. 2021. [cited 2024]. Available from: https://www.esri.com/arcgis-blog/products/arcgis-pro/analytics/looking-to-the-future-using-gis-to-model-and-predict-population/.

56. Fuenfschilling L, Binz C. Global socio-technical regimes. Research Policy. 2018;47(4):735-49.

57. Fuenfschilling L, Truffer B. The structuration of socio-technical regimes—Conceptual foundations from institutional theory. Research Policy. 2014;43(4):772-91.

58. Kimari W. We will be back to the street!': Protest and the 'empires' of water in Nairobi. In: Bekker S, Croese S, Pieterse E, editors. Refractions of the National, the Popular and the Global in African Cities. Cape Town, South Africa: AfricanMinds; 2021. p. 99-110.

59. Scott P, Cotton AP. The Sanitation Cityscape – Toward a Conceptual Framework for Integrated and Citywide Urban Sanitation. Frontiers in Environmental Science. 2020;8.

60. Mutavi L. Where does your 'shit' go? 66% of Nairobi human waste unaccounted for. The Star. 2018 05 July 2018.

61. Mallory A, Omoga L, Kiogora D, Riungu J, Kagendi D, Parker A. Understanding the role of informal pit emptiers in sanitation in Nairobi through case studies in Mukuru and Kibera settlements. Journal of Water, Sanitation and Hygiene for Development. 2021;11(1):51-9.

62. Muindi K, Mberu B, Aboderin I, Amugsi D. Conflict and crime in municipal solid waste management: evidence from Mombasa and Nairobi, Kenya. Cities & Health. 2022;6(1):159-67.

63. Kareithi A. Faces behind city suburbs shitload of sewer millions. The Standard. 2020.

64. Anciano F, Dube M, Mdee A, Riungu J, Karani C. The Moral Economy of Sanitation Provision in Informal Settlements in Cape Town and Nairobi. forthcoming.

65. The World Bank. Evaluating the potential of container-based sanitation: Sanergy in Nairobi, Kenya. Washington, DC: The World Bank; 2019.

66. Mwau B, Sverdlik A. High rises and low-quality shelter: rental housing dynamics in Mathare Valley, Nairobi. 2020;32(2):481-502.

67. Evans B, Hueso A, Johnston R, Norman G, Pérez E, Slaymaker T, et al. Limited services? The role of shared sanitation in the 2030 Agenda for Sustainable Development. Journal of Water, Sanitation and Hygiene for Development. 2017;7(3):349-51.

68. Heijnen M, Cumming O, Peletz R, Chan GK-S, Brown J, Baker K, et al. Shared Sanitation versus Individual Household Latrines: A Systematic Review of Health Outcomes. PLOS ONE. 2014;9(4):e93300.

69. Foggitt E, Cawood S, Evans B, Acheampong P. Experiences of shared sanitation – towards a better understanding of access, exclusion and 'toilet mobility' in low-income urban areas. Journal of Water, Sanitation and Hygiene for Development. 2019;9(3):581-90.

70. Integrated Urban Development Master Plan for the City of Nairobi, (2014).

71. AfDB. Nairobi Rivers Basin Rehabilitation And Restoration Program: Sewerage Improvement Project Phase II. Project Appraisal Report. African Development Bank (AfDB); 2018.

72. Catapult. Nairobi: Solid Waste Management & Storm Water Drainage and Flooding: Catapult; 2021.

73. WHO and UNICEF. JMP Monitoring: Sanitation: WHO and UNICEF; 2024 [Available from: https://washdata.org/monitoring/sanitation.

74. WASREB. Impact 14. A performance report of Kenya's Water Services Sector 2020/21. Nairobi, Kenya: Water Services Regulatory Board; 2022.

75. KNBS and ICF. Kenya Demographic and Health Survey 2022: Volume 1. Nairobi, Kenya, and Rockville, Maryland, USA: KNBS and ICF; 2023.

76. Corburn J, Hildebrand C. Slum Sanitation and the Social Determinants of Women's Health in Nairobi, Kenya. Journal of Environmental and Public Health. 2015;2015:1-6.

77. World Bank. Country Climate and Development Report: Kenya - Urban Sector Background Note. Washington DC, USA: The World Bank; 2023.

78. Galvin M, Maassen A. In Nairobi, residents redesign public spaces to build flood resilience: World Resources Institute; 2021 [Available from: https://www.wri.org/nairobi-public-spaces-build-flood-resilience.

79. Kinyanjui M. Heavy rains leave Nairobi estates, roads flooded. The Star. 2023 23 July 2023.

80. Njaaga D. Nairobians brave flooded roads after night of heavy downpour. The Standard. 2023 23 July 2023.

81. Vane CH, Kim AW, Lopes dos Santos RA, Gill JC, Moss-Hayes V, Mulu JK, et al. Impact of organic pollutants from urban slum informal settlements on sustainable development goals and river sediment quality, Nairobi, Kenya, Africa. Applied Geochemistry. 2022;146:105468.

82. Ward PJ, Jongman B, Salamon P, Simpson A, Bates P, De Groeve T, et al. Usefulness and limitations of global flood risk models. Nature Climate Change. 2015;5(8):712-5.

83. Nkwunonwo UC, Whitworth M, Baily B. A review of the current status of flood modelling for urban flood risk management in the developing countries. Scientific African. 2020;7:e00269.

84. Randa TO, Krhoda GO, Atela JO, Akala H. Review of flood modelling and models in developing cities and informal settlements: A case of Nairobi city. Journal of Hydrology: Regional Studies. 2022;43:101188.

85. The World Bank. Kenya Urbanization Review. Washington DC, USA: The World Bank; 2016.

86. Ominde J. ASSESSING LAND USE AND LAND COVER CHANGE IMPACT ON FLASH FLOODS IN KASARANI CONSTITUENCY, KENYA. IJRDO - Journal of Social Science and Humanities Research. 2022;8(10):1-17.

87. NCWSC. Strategic Plan 2019/20-2023/24. Nairobi, Kenya: Nairobi City Water and Sewerage Company Limited; 2019.

88. Kasper M, Schramm S. Storage city: Water tanks, jerry cans, and batteries as infrastructure in Nairobi. 2023;60(12):2400-17.

89. Mati E, Owako E. A guide to simplified sewer systems in Kenya. Nairobi, Kenya: Water and Sanitation for the Urban Poor (WSUP); 2022.

90. Weinstein L, Rumbach A, Sinha S. Resilient Growth: Fantasy Plans and Unplanned Developments in India's Flood-Prone Coastal Cities. 2019;43(2):273-91.

91. Watson V. African urban fantasies: dreams or nightmares? Environment and Urbanization. 2014;26(1):215-31.

92. RoK. The Climate Change Act. In: Kenya Ro, editor. Nairobi, Kenya: Republic of Kenya; 2016.

93. NCC. County Integrated Development Plan 2023-2027. In: County NC, editor. Nairobi, Kenya: Nairobi City County; 2023.

94. MoEF. Nationally Determined Contribution: Kenya. 2020 update. In: Forestry MoEa, editor. Nairobi, Kenya: Republic of Kenya; 2020.

95. MoENR. Kenya National Adaptation Plan 2015-2030. In: Resources MoEaN, editor. Nairobi, Kenya: Republic of Kenya; 2016.

96. MoEWNR and WRMA. The National Water Master Plan 2030. In: Ministry of Environment WaNRWRMA, editor. Nairobi, Kenya: Republic of Kenya; 2013.

97. MoWSI. National Water and Sanitation Strategy 2020-2025. In: Ministry of Water SaI, editor. Nairobi, Kenya: Republic of Kenya; 2021.

98. MoWSI. Development of the National Sanitation Management Policy (NSMP) in Kenya. A narrative descriptive account of the journey. In: Ministry of Water SaI, editor. Nairobi, Kenya: Republic of Kenya; 2022.

99. GoK, CPI, KCIC. The Landscape of Climate Finance in Kenya. On the road to implementing Kenya's NDC. Nairobi, Kenya: The National Treasury, Climate Policy Initiative and the Kenya Climate Innovation Centre; 2021.

100. County Annual Development Plan (CADP) 2022/23, (2021).

101. GoK. Water Act. In: Kenya GotRo, editor. Nairobi: Government of the Republic of Kenya; 2016.

102. AWWDA. Strategic Plan 2018-2022. Nairobi, Kenya: Athi Water Works Development Agency; 2018.

103. MoWSI. Financing Water Sector in Kenya: Ministry of Water, Sanitation and Irrigation; 2019 [Available from: https://www.waterreforms.go.ke/financing-water-sector-in-kenya/#:~:text=Water%20sector%20is%20heavily%20donor,private%20sector%20playing%20negligible%20role.

104. The World Bank. Kenya - Urban Support Program: Project Appraisal Document. Washington DC: The World Bank; 2017.

105. The World Bank. Kenya - Second Kenya Informal Settlements Improvement Project: Project Appraisal Report. Washington DC: The World Bank; 2020.

106. Kamunyori S. Disclosable Version of the ISR - Second Kenya Informal Settlements Improvement Project - P167814 - Sequence No : 06 Washington DC: The World Bank; 2023.

107. AWF. Nairobi Inclusive Sanitation Improvement Project. Appraisal Report. Abidijan, Cote d'Ivoire: African Water Facility (AWF); 2019.

108. GCF. Funding Proposal: FP205: Infrastructure Climate Resilient Fund (ICRF). Green Climate Fund; 2023.

109. Dodman D, Satterthwaite D. Institutional capacity, climate change adaptation and the urban poor. IDS; 2008.

110. Shi L, Chu E, Anguelovski I, Aylett A, Debats J, Goh K, et al. Roadmap towards justice in urban climate adaptation research. Nature Climate Change. 2016;6(2):131-7.

111. MoWI. The Pro-Poor Implementation Plan for Water Supply and Sanitation (PPIP - WSS). Nairobi, Kenya: Ministry of Water and Irrigation; 2007.

112. Werchota R. Empty Buckets and Overflowing Pits. Urban Water and Sanitation Reforms in Sub-Saharan Arica - Acknowledging Decline, Preparing for the Unprecedented Wave of Demand. Kostianoy AG, editor. Cham, Switzerland: Springer Nature Switzerland AG; 2020.

113. WASREB. A Performance Report of Kenya's Water Services Sector 2015/16 and 2016/17. Nairobi, Kenya: Water Services Regulatory Board; 2018.

114. WASREB. Guidelines on Sanitation Levy And Trade Effluent Surcharge by Water Service Providers in Kenya. Nairobi, Kenya: Water Services Regulatory Board; 2019.

115. Reuters. Kenyan court strikes down housing levy, stays ruling until Jan 10. Reuters. 2023 29 November 2023.

116. GoK. National Climate Change Response Strategy. Nairobi, Kenya: Government of Kenya; 2010.

117. GoK. National Climate Change Action Plan (Kenya) 2018-2022. Nairobi, Kenya: Ministry of Environment and Forestry; 2018.

118. Monstadt J, Schramm S. Toward The Networked City? Translating Technological ideals and Planning Models in Water and Sanitation Systems in Dar es Salaam. International Journal of Urban and Regional Research. 2017;41(1):104-25.

119. Igarashi J, Evans B, Sleigh A, Tarkash DN, Kennedy R, Rosenberg R, et al. Does "Low Cost" Urban Sanitation Exist? Lessons from a Global Data Set. Environmental Science & Technology. 2023;57(45):17237-45.

120. The World Bank. Kenya Countywide Inclusive Sanitation Strategic Planning: Guidance Note. Nairobi, Kenya: The World Bank and Republic of Kenya; 2019.

121. Brugger A. Unbundling the urban sanitation crisis: a territorial history of the development of urban sanitation. MAPS Working Paper Series: The Circulation of Wealth 2021;2(2-2021/E):1-35.

122. Williams J. “Money is Not the Problem”: The Slow Financialisation of Kenya’s Water Sector. Antipode. 2021;53(6):1873-94.

123. Heckel M. Water utilities as debt emitters: the commercialization of development funding and services provision in Kenya’s water sector. Globalizations.1-19.

124. GoK. Third Medium Term Plan 2018 – 2022: Transforming Lives: Advancing socio-economic development through the “Big Four”: Government of the Republic of Kenya; 2018.

125. Horn P. Enabling participatory planning to be scaled in exclusionary urban political environments: lessons from the Mukuru Special Planning Area in Nairobi. Environment and Urbanization. 2021;33(2):519-38.

126. Bassett E. Reform and resistance: The political economy of land and planning reform in Kenya. 2020;57(6):1164-83.

127. Ouma S, Corburn J, Weru J. Property rights, public health, and planning for informal settlements in Nairobi, Kenya. Journal of Urban Affairs. 2023;45(3):302-16.

128. Schramm S, Ibrahim B. Hacking the pipes: Hydro-political currents in a Nairobi housing estate. 2021;39(2):354-70.

129. Corburn J. Water and sanitation for all: Citizen science, health equity, and urban climate justice. Environment and Planning B: Urban Analytics and City Science. 2022;49(8):2044-53.

130. Greibe Andersen J, Kallestrup P, Karekezi C, Yonga G, Kraef C. Climate change and health risks in Mukuru informal settlement in Nairobi, Kenya – knowledge, attitudes and practices among residents. BMC Public Health. 2023;23(1).

131. Ouma S. Participation as ‘city-making’: a critical assessment of participatory planning in the Mukuru Special Planning Area in Nairobi, Kenya. Environment and Urbanization. 2023;35(2):470-89.

132. UN Habitat. Kenya UN Habitat Country Programme Document 2018-2021: Enhancing effective service delivery and sustainable urban development at national and county levels. Nairobi: UN Habitat; 2018.

133. The World Bank. Kenya Urban Support Program 2023 [cited 2023. Available from: https://projects.worldbank.org/en/projects-operations/project-detail/P156777.

134. U.S. Census Bureau. Kenya Annual Five-Year Age Group Population Estimates by Sex for 2015 to 2030: National, and First- and Second-Order Administrative Divisions. In: U.S. Census Bureau PD, editor. 2023.

135. Kennedy-Walker R, Mehta N, Thomas S, Gambrill M. Connecting the Unconnected: Approaches for Getting Households to Connect to Sewerage Network. Washington DC, USA: The World Bank; 2020.

136. Heath TT, Parker AH, Weatherhead EK. Testing a rapid climate change adaptation assessment for water and sanitation providers in informal settlements in three cities in sub-Saharan Africa. Environment and Urbanization. 2012;24(2):619-37.

137. Alam SS, Alam AJ, Rahman S. Urban climate resilience, water and sanitation: improving multi-stakeholder collaboration in Dhaka, Bangladesh. London; 2015.

138. Purwar D, Sliuzas R, Flacke J. Assessment of cascading effects of typhoons on water and sanitation services: A case study of informal settlements in Malabon, Philippines. International Journal of Disaster Risk Reduction. 2020;51.

139. Shimi AC, Parvin GA, Biswas C, Shaw R. Impact and adaptation to flood: A focus on water supply, sanitation and health problems of rural community in Bangladesh. Disaster Prevention and Management: An International Journal. 2010;19(3):298-313.

140. Gathanga S. Limitations of the NGO discourse. In: Mwangi N, Maghanga L, editors. Breaking the Silence on NGOs in Africa. Wakefield, Québec, Canada: Daraja Press; 2023. p. 58-60.

Supporting information

S1 Table. Summary of reviewed Climate Change Policy and Planning Framework Documents with focus on specific references to sanitation system adaptation and equity considerations

S2Table. Summary of reviewed Sanitation Sector Policy and Planning Framework Documents with focus on specific references to climate change adaptation and equity considerations

S3Table. Summary of reviewed sanitation investments in Nairobi county with focus on specific references to sanitation system adaptation and equity considerations

S4 Annex. Description of population forecasting method using ArcGIS Pro time series forecasting tools

Figure 2 Click here to access/download;Figure;Fig 2.tif

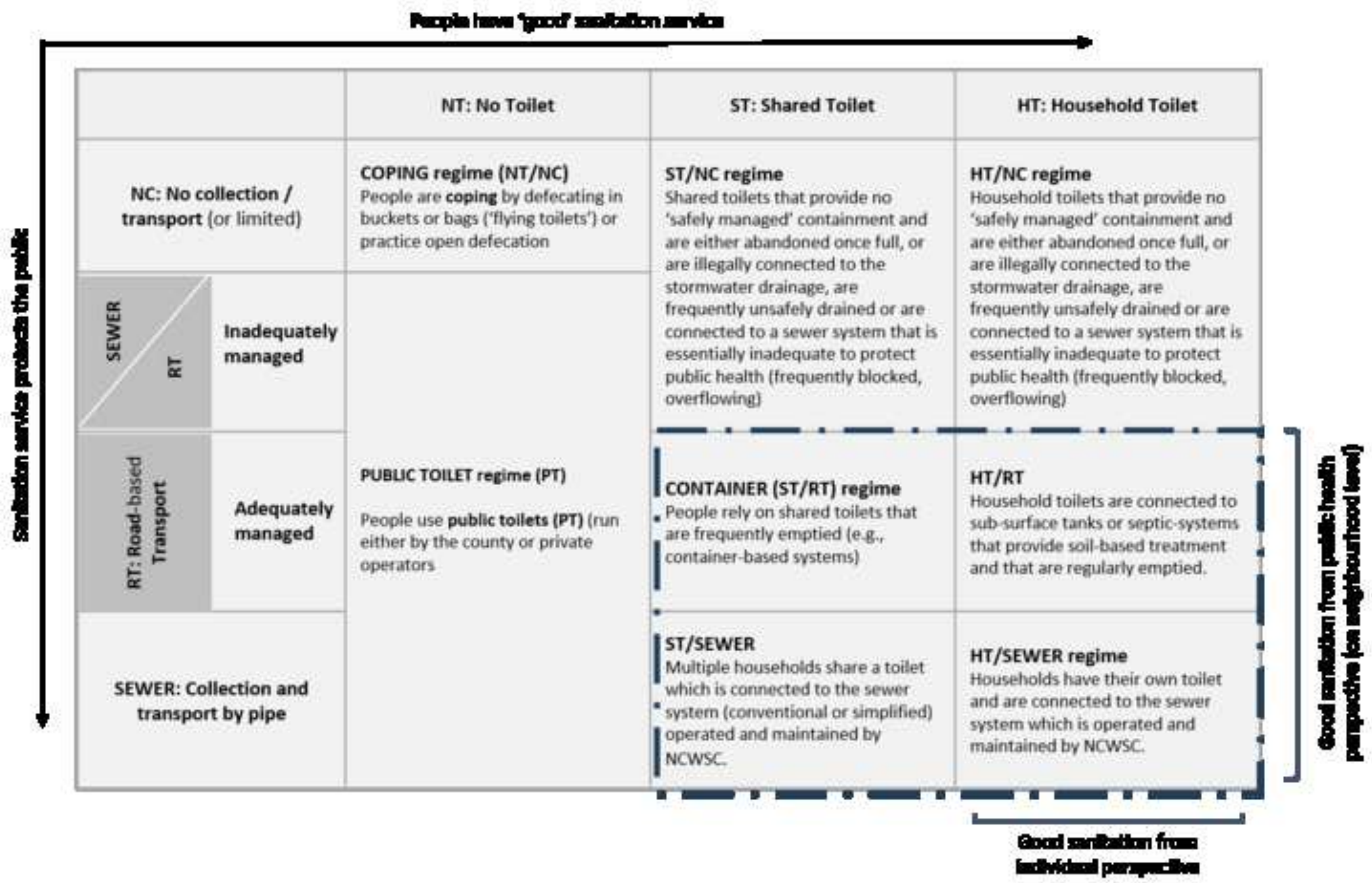

Figure 3

Click here to access/download;Figure;Fig 3.tif

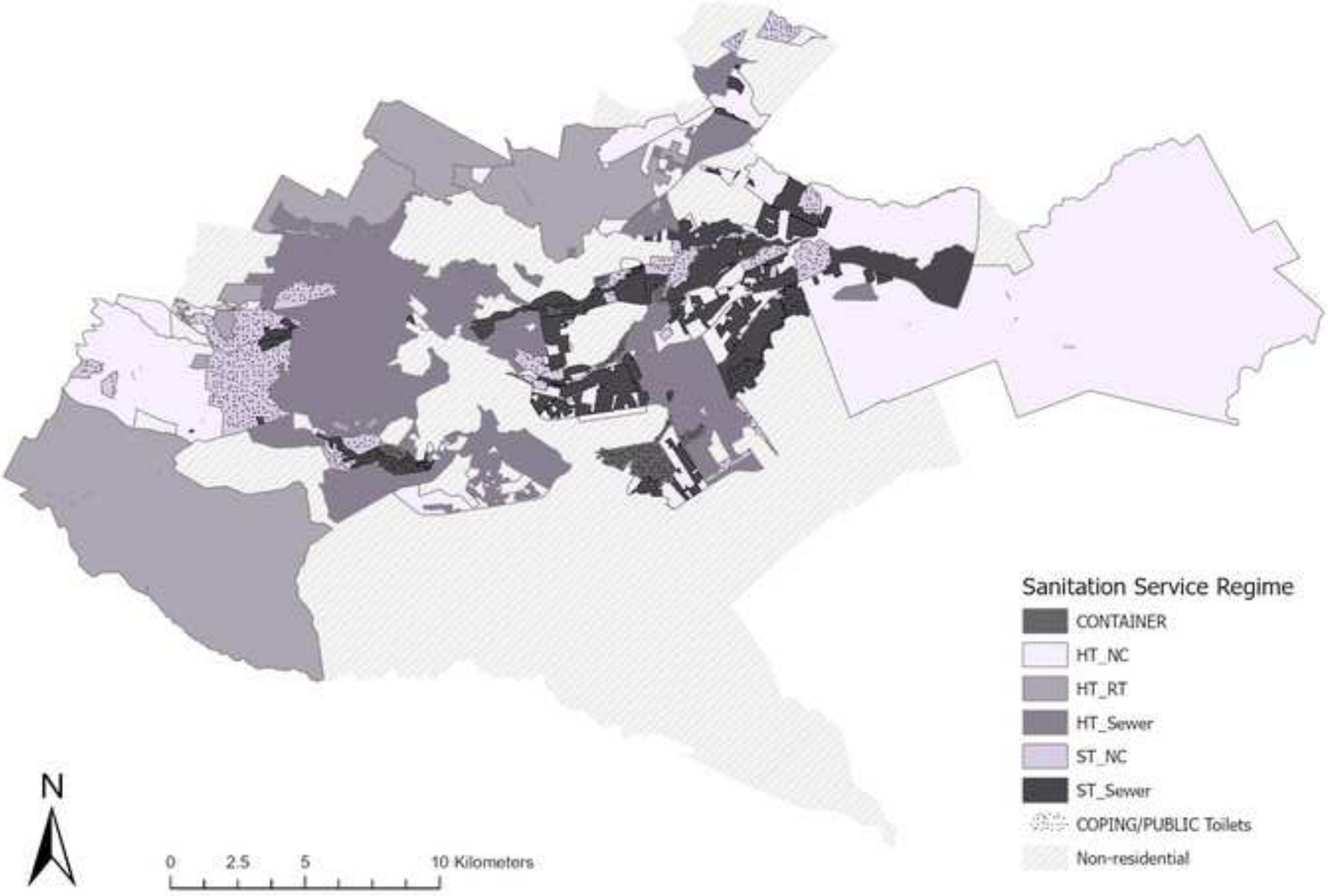

Figure 4

Click here to access/download;Figure;Fig 4.tif

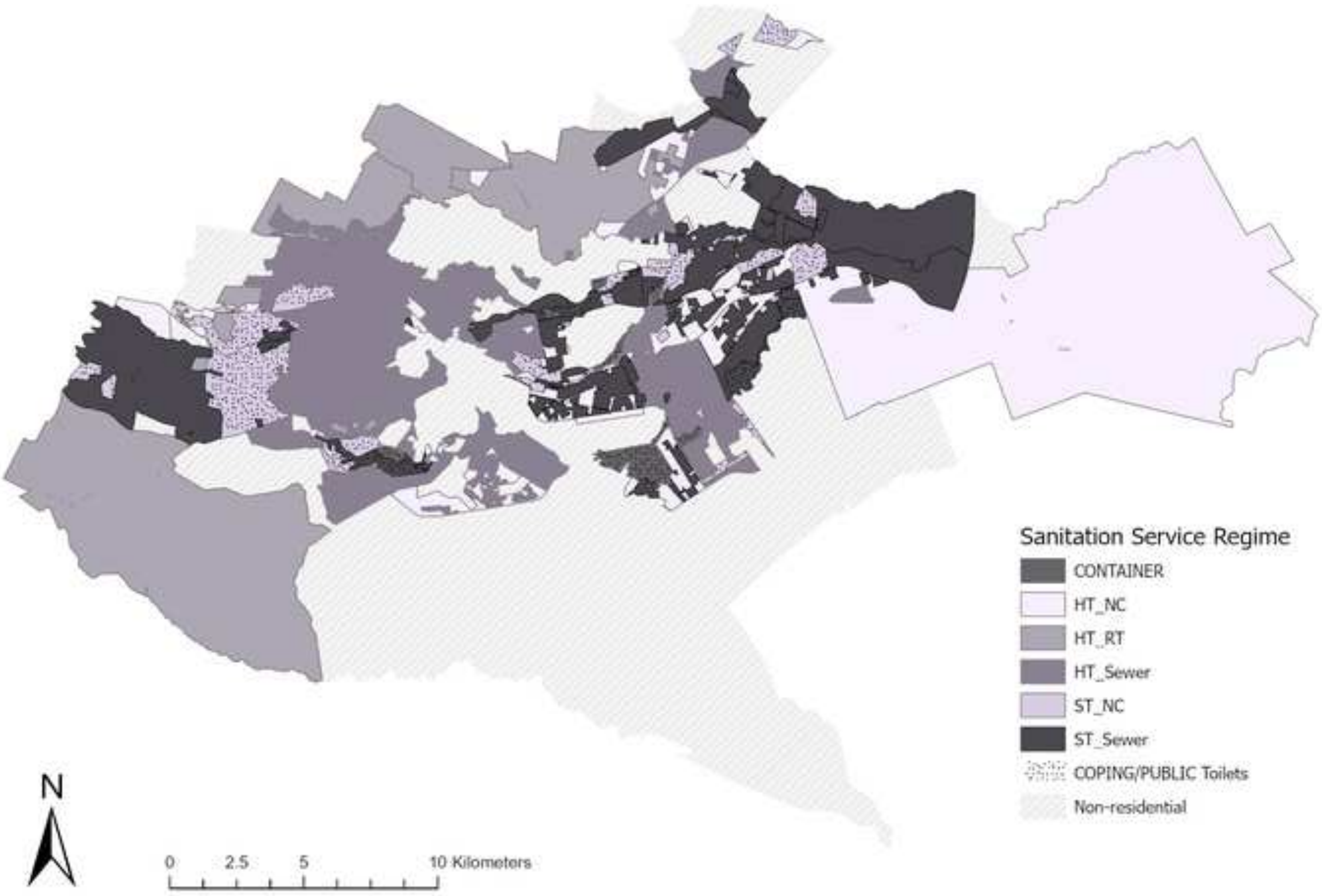

Figure 1

Click here to access/download;Figure;Fig 1.tiff

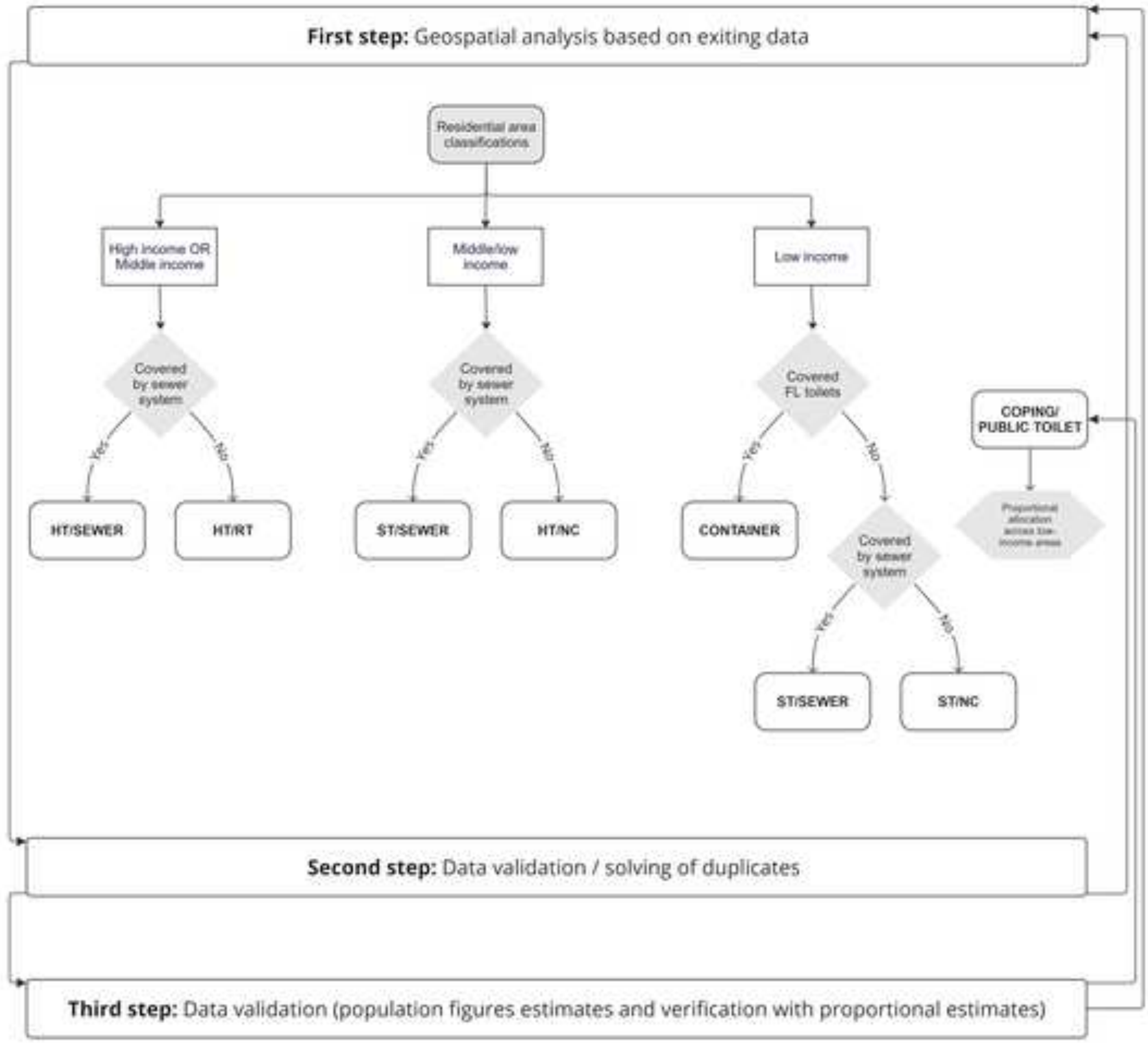

COPING (NC/NT) = People don't have access to toilets us coping mechanisms; PUBLIC TOILET = People rely on public toilets as main form of sanitation; ST/NC = Shared toilets that are NOT adequately managed; CONTAINER (ST/RT) = Shared toilets that are regularly emptied; ST/SEWER = Shared toilets connected to the sewer system; HT/NC = Household that are not adequately managed; HT/RT = Household toilets that regularly emptied; HT/SEWER = Household toilets connected to the sewer system

Click here to access/download
Supporting Information
S1 Table.pdf

Click here to access/download

Supporting Information

S2 Table.pdf

Click here to access/download
Supporting Information
S3 Table.pdf

Click here to access/download
Supporting Information
S4 Annex.pdf
