# Supplementary material for: Business-as-usual and fantasy planning – an analysis of equity within climate adaptation planning for sanitation in Nairobi
Source: PLoS One. 2025 Dec 30;20(12):e0339272. doi: 10.1371/journal.pone.0339272 (PMC12752985; doi:10.1371/journal.pone.0339272)
Supplement: Attachment — Submitted filename: CC Sanitation Equity Nairobi_comment_response matrix.docx [file pone.0339272.s006.docx]

| **No** | **Reviewer** | **Section / Line** | **Comment** | **Response** |
| --- | --- | --- | --- | --- |
| 1. | Rev1 | General | Overall, the paper contributed to the body of knowledge on the intersections of climate change, urban sanitation, and service equity, with broader implications for policymakers, practitioners, and researchers working in this field. | Thank you. |
| 2. | Rev1 | Introduction | The introduction was well-written and effectively set the scene for the paper. However, starting the introduction with a numbered statement might not be the most appropriate approach. The introduction could further benefit from providing the readers with a clear articulation of this paper's unique contribution to the body of knowledge. | We have further clarified the aim/contribution of the paper as *This sudy aims to examine the disparities between proclaimed and actual equity considerations at the intersection of urban sanitation planning and climate change adaptation in a city characterised by substanital sanitation inequalities in a resource constraint setting* (p.4 line 10-104)*.* |
| 3. | Rev1 | Introduction | The introduction should mention the broader significance and applicability of the research beyond the Nairobi case study, indicating the relevance to other urban centers facing similar challenges. | See revised overall study aim (**response above**) |
| 4. | Rev1 | Methodology | The authors mention that the modeled changes in flood depth and extent between 2020, 2030, and 2050 showed only minor changes. This raises questions about the robustness of the flood risk assessment and whether it adequately captures the potential impacts of climate change on the sanitation system. | We agree that this section needed clarification. Detailed flood modelling was beyond the scope of the study and therefore our ‘results’ lacked validity. We have revised the section accordingly. |
| 5. | Rev1 | Methodology | The authors do not provide detailed information on the specific data sources, methods, and assumptions used in the geospatial analysis to map the different types of sanitation systems. This lack of transparency may limit the replicability and evaluation of the approach. | We provide the data sources of our shape files, satellite images and income specification.   - Mutono N, Wright J, Mutembei H, Thumbi SM. Spatio-temporal patterns of domestic water distribution, consumption and sufficiency: Neighbourhood inequalities in Nairobi, Kenya. Habitat International. 2022;119:102476. - Ledant M. Access to Water in Nairobi: mapping inequalities beyond statistics. In: UN-Habitat Ga, editor. 2013. - Google, cartographer Satellite imaginary of Nairobi2023.   We have added to the decription of validation (see response to comment 34  … to verify consistency and detect any changes in land use or income classification patterns. Key indicators, such as visible infrastructure developments, road networks, and urban expansion, were cross-checked to ensure the shapefiles matched current urban layouts.  Figure 1 decribes the mapping and categorisation process |
| 6. | Rev1 | Results | While the results highlight the persistent gaps between policy aspirations and practical implementation, especially in the critical areas of urban planning, climate resilience, and sanitation service delivery, the analysis could delve deeper into the underlying reasons for the disconnect between policy and implementation. | Thank you we have added a summary paragraph addressing some the underlying issues: In summary, in Nairobi, the sanitation policy and planning framework mimic ‘recognition’ of sanitation inequities and climate threats whilst failing to translate their ambitious and performative statements into actions that would genuinely enhance the procedural or distributive equity within the ‘splintered sanitation sector’. Additionally, national policies in Kenya extravert ‘pro-poor’ inclusion within the water and sanitation sector as well as climate change mainstreaming. This is emblematic of a broader trend where development partners use aid conditions to incentivise governments to reproduce ‘best practice’ reforms of public sectors – such as the water sector – fostering the establishment of ostensibly ‘ideal’ institutions equipped with symbolic ‘good’ policy and institutional frameworks whilst lacking the requisite capability to fulfil their promises. These findings echo Narzetti and Marques {Narzetti, 2021 #6}analysis of reforms of Brazilian water and sanitation who describe the ineffectiveness of the de jure well designed ‘pro-poor’ and ‘inclusive’ water sector policy framework that de facto fails the 100 million Brazilians without wastewater collection services and largely excludes the peri-urban population. |
| 7. | Rev1 | Results | The analysis could also draw parallels or contrasts with how the policy-implementation gaps manifest in other sectors. | We refer to flood resilience planning in Mumbai (p.20 lin 133 ff) |
| 8. | Rev1 | Conclusion | The conclusion could include specific recommendations or a call to action for policymakers, government officials, development partners, and other stakeholders to address the identified gaps and challenges. | We have revised the last paragraph of the conclusion to address this comment: *Our analysis, therefore, underscores the urgent need for a fundamental shift in approach. Placing city governments at the centre of co-production processes for sanitation adaptation strategy (141) may offer effective paths towards adaptation plans that are more grounded in reality and go beyond fantasy planning. Further research in this area would be highly valuable. In addition, there must be an honest and robust discussion about providing high-quality, resilient services at scale beyond sewered areas, necessitating substantial government funding and support.* |
| 9. | Rev1 | Conclusion | The conclusion could mention potential areas for future research that could build on the findings of this analysis. | See above |
| 10. | Rev2 | General | An worthwhile paper but needs to be refined with a clear purpose and aim. Reference to frameworks, methods and analytical results need more attention and focus. The conclusion raises at least 2 critical points that are impactful and need further explanation and support in the preceding discussion. See comments on attached file. | Thank you. See our responses to the detailed comments below |
| 11. | Rev 2 | Abstract | Rewrite the abstract. Single paragraph. Include statements of method, main findings that can be briefly qualified, conclusion and recommendation (if any). | Abstract was revised |
| 12. | Rev2 | Abstract | Rewrite the abstract. Single paragraph. Include statements of method, main findings that can be briefly qualified, conclusion and recommendation (if any) | Abstract was revised |
| 13. | Rev2 | Abstract | Qualify this statement: in the South this disjuncture between rhetoric and implementation is a common feature. Highlight a particular finding that qualifies this observation. The sentence that follows may be the qualification but it is unclear. | Wording changed |
| 14. |  | Abstract | Consider removing or qualifying the adjectives or pronouns that appear these final sentences that 'oversell' or exaggerate the conclusions. e.g. earnest discussion, fundamental, critical, substantial etc. Be clear about the conclusion and the recommendations if any. | Wording changed |
| 15. |  | Introduction p.3 line58 | Explain and consider using this to explain the title of the paper: what you intend to discuss in the using the words 'business as usual' and fantasy planning. Bring these ideas in earlier. | Introduced terms in introduction |
| 16. |  | Introduction p.3 line62 | Explain...there are many such frameworks with different emphases. | Changed to … assessed through a justice lense… We give an explanation of the conceptualisation of climate adaptation justice in the following sentences |
| 17. |  | Introduction p.3 line69 f | This is an important statement and should be the crux of the paragraph for developing the focus of the paper. | Thank you. We have further clarified the aim of the paper see response to **reviewer comment 2** |
| 18. |  | Introduction p.3 line73 | A good reason, but it limits the scope and generalisations that could be achieved from the paper. | We have revised the section and think it is now clearer linked to the overall aim of the paper. Nairobi is used as an illustrative case for a city with severe sanitation inequalities while we also accept that there are limitations to generalisations from a distinct city case. |
| 19. |  | Introduction p.3 line75 | Meaning obscure at this stage of the paper | We have revised the section for more clarity |
| 20. |  | Introduction p.3 line77 | Lots of jargon in this long sentence. Explain in more detail. | We have revised the section for more clarity |
| 21. |  | Introduction p.4 line80 | Referring to what? | We have revised the section for more clarity |
| 22. |  | Introduction p.4 line80 | Framework needs further explanation | We have revised the section for more clarity |
| 23. |  | Introduction p.4 line81 | Still too vague/confusing. Simplify. | We have revised the section for more clarity |
| 24. |  | Introduction p.4 line82 | What are these? Aligned with what? | We have revised the section for more clarity |
| 25. |  | Introduction p.4 line86 | Landscape? Sector? Regime? Lots of concepts that are left unexplained and result in confusion. | We have revised the section for more clarity |
| 26. |  | Introduction p.4 line89 | Explain. The lack of services, and flying toilets are not necessarily able to explain cause and effect relations, but are context specific in a social-economic setting. The reference may need more explanation. | We have revised the section for more clarity |
| 27. |  | Introduction p.4 line94 | ...and it certainly does, but is unable to reach the scale that this required. There are many innovative examples and efforts in Nairobi. | Agreed – we have revised to acknowledge the initiatives : While there have been numerous applaudable and innovative sanitation efforts in Nairobi reflecting this commitment, these initiatives have struggled to achieve the scale necessary to effectively address the needs of marginalized populations. |
| 28. |  | Introduction p.4 line100 | Before presenting the objectives, it will be important to clarify the overall aim and purpose of the paper (linked to the title?). | Thank you. We have further clarified the aim of the paper see response to **reviewer comment 2** |
| 29. |  | Introduction p.5 line106 | Needs to be explained in the preceding introduction. | We have introduced the three common tenets of environmental / adaptation justice in the preceeding introduction and give a detailed interpretation of these in the context of sanitation adaptation in Table 2 |
| 30. |  | Introduction p.5 line107f | Perhaps too speculative? | This is based on the results of a systematic review on climate impacts on sanitation failures as well as on climate scenarios that were modelled under the Hycristal project in collaboration with the UK Met Office. We therefore belive that our projections are grounded in robust data. |
| 31. |  | Introduction p.5 line109ff | This objective needs further refinement. It appears to be a task, but it is unclear what is going to be achieved. | Revised accordingly: Evaluate the adequacy of Nairobi’s policy and planning frameworks in addressing the additional pressures posed by climate change on sanitation systems. This involves assessing whether and how climate adaptation is incorporated into the city’s sanitation planning and investment framework, and conversely, whether sanitation considerations are integrated into Nairobi’s broader climate adaptation planning and investment framework. The aim is to determine whether these frameworks are fit-for-purpose to prevent increased vulnerabilities and inequalities resulting from potential climate hazard related sanitation system failures |
| 32. |  | Introduction p.5 line112f | A duplication of objective #2? Explain. | We changed the wording of the objective for more clarity. The difference to objective 2 is that objective assesses the likely impacts on the different sanitation service regimes on a theoretical level whereas objective 4 attempts to quantify the effects in terms of distributional equity |
| 33. |  | Method p.5 line117f | What is this framework? What are indicators or metrics to be used? | We describe the classifications and mapping process in Figure 1 |
| 34. |  | Method p.5 line121 | How was this achieved? Are there method statements? | We added a further description … to verify consistency and detect any changes in land use or income classification patterns. Key indicators, such as visible infrastructure developments, road networks, and urban expansion, were cross-checked to ensure the shapefiles matched current urban layouts. |
| 35. |  | Method p.5 line122 | What is this 'rapid' growth? Discussion missing. | Added reference |
| 36. |  | Method p.5 line126 | Will these sources, their validation and credibility be explained? Is the evidence robust? It is difficult to determine. | We give the source of a recent base map and of the sanitation data (see also responses to **comments 5 and 58** |
| 37. |  | Method p.6 Figure 1 | A poor map. Difficult to distinguish the categories. The table of explanation is blurred. A high resolution, colour map and a table of categories is recommended. | Hi-res image included |
| 38. |  | Table 1 p6/7 | Explain how Future 3 contradicts(?) 1 and 2. | Future 3 is most extreme in terms of warming and also linked to erratic rainy seasons and prolonged dry spelts and droughts |
| 39. |  | p.7 line 156 | Explain and why the selection was made | See response to comment 41 |
| 40. |  | p.7 line 158 | unsubstantiated? | See response to comment 41 |
| 41. |  | p.8 line 161 | Difficult to understand the value of this discussion section and purpose since it remains speculative and unsubstantiated. | We agree that this section was confusing. The section has been removed. See response to **reviewer comment 4** |
| 42. |  | p.7 line 179 | So, multiple sources were consulted. The method of extraction, indicators and framework were not discussed in any detail. This should be included in a discussion. | Our thematic analysis is described in the following paragraph and Table 2 gives our analysis framework / evaluation critieria |
| 43. |  | p.8 line 186 | Adapted? In what way? Does the discussion in the previous paragraph advance the adaptation of Wood and Roelich? In what way? Is there a connection? It reads like an illogical link. | We based our framing of sanitation adaptation justice on Wood and Roelich’s exploration of energy justice and adapted it to the sanitation sector |
| 44. |  | p.8 line 179 | which are what? Clarify | Reformulated for clarity |
| 45. |  | p.9 line 198 (reference to S4 Annex) | Not available to the reviewer. The process in developing these projections is unclear and cannot be verified from a short descriptive paragraph. | The forecasting method is described in detail in the Supplementary Material |
| 46. |  | p.9 line201 | Redundant sentence | We believe that this sentence has value as sanitation is still understood as mainly infrastructure by many |
| 47. |  | p. 9 line 202 | Still underexplained since there are many frameworks and concepts of so called socio-technical regimes. | We moved this section to the introduction and revised the corresponding section |
| 48. |  | p.9 line 204 | Sure, but what is the position and conceptual understanding of the authors and how does this connect to the aim, purpose and objectives of the paper? | See above |
| 49. |  | p.9 line 211 | Sure, but this is an uncritical approach to context and success of informal and decentralised systems in a 'chaotic' densely settled peri urban city. Its much more complex and that centralised systems are not the only 'solution'. | We fully agree on the point made about centralised systems not being the only solution. This is also reflected in our assessment of distributional equity shown in figure 2 which acknowledges the role of full service chain decentralised solutions as part of an adequate sanitation service provision. Unfortunately we understand from comment 51 that the reviewer was not able to access the figure. |
| 50. |  | p.10 line 221 | Sure, shows some recognition of the scope and limitations of the study and thinking. Good to read this? | OK |
| 51. |  | p.10 line 223 (Figure 2) | Not available to reviewer. All links in Annexure were not functional | OK |
| 52. |  | p.10 line 227 | Who are these residents? Is there empirical evidence and a method? | Reference added |
| 53. |  | p.10 line 232 | Evidence or a reference? | Reference added |
| 54. |  | P.11 line 264 | The scale changes again from the discussion in the preceding section. What is the scale and unit of measurement that is central to the paper and in making comparison for the sake of inequity within the city? Assuming that this remains a central purpose of the paper. | We analyse the sanitation system in Nairobi throughout the discussion section. We also refer to the legal and policy framework in Kenya as a country but we do not understand the comment or what has caused the confusion about the scale |
| 55. |  | p.12 line 279 | This is broad statement that needs to be verified. It is likely that these systems are unregulated and cause harm to groundwater sources. | We refer to the unregulated design standards and environmental risk in the next sentence. We mainly want to state that the public health risk from these facilities is likely to be transferred from the user to the wider community |
| 56. |  | p.12 line 283 | Agreed and in relation to my previous comment. | OK |
| 57. |  | p.12 line 291 (Figure 3) | Poor image - blurred and not easy to read | Updated High-res image provide |
| 58. |  | p.13 line 297 | Validation methods are inadequately explained. | We mention our data sources which is regulator data, census and published and peer-reviewed sanitation data |
| 59. |  | p.14 line 325 | Given a host of known issue in Nairobi, it is worth considering how to improve this paper dealing with these issues including an interrogation of governance, policy and financial resources, rather than introduce climate change as another factor that detracts from the management issues on the ground. As a suggestion.... | See response below. We acknowledge that there is a host of other sanitation related challenges in Nairobi. Our paper however concentrates on climate adapation as we state in the introduction: *There is growing recognition that climate-adaptive sanitation infrastructure is non-negotiable for avoiding the risk of reversing progress towards universal access to safe sanitation services (13, 14) and thereby worsening inequalities. However, attention to adaptation needs within urban sanitation systems has been limited and primarily focused on technology solutions (15). Sanitation adaptation is mainly happening in high-income countries, focusing on retrofitting and upgrading old infrastructure approaching the end of its design life (16, 17).* |
| 60. |  | p.15 Table 4 | These scenarios are speculative and detract from the critical point that emerges here about sanitation management and adaptation to circumstances. | See our response to **comment 30 –** the climate scenarios and impact analysis is based on robust research. Climate adaptation of sanitation systems is at the heart of this paper and we have strengthened the climate framing in the introduction |
| 61. |  | p. 18 line 46 | Another section... The paper is fragmented by sections rather than depth, purpose, method explanations, and analysis of empirical data. Consider omitting sections. While policy is an important factor, it is introduced here as an other element that detracts from an aim. | We structured our discussion section along our research objectives that as a sum contribute to the overall study aim |
| 62. |  | p.18 line 48 | Why not embed it in the gap in Nairobi? Scale changes again? | As stated in the paragraph above, we embed our discussion within the broader debate on the gaps between policy and implementation. Nairobi is illustrative for a city, where ambitious policies often face challenges in practical execution due to resource constraints, institutional inefficiencies, and competing priorities. In the context of climate adaptation, such gaps can be compounded by the risk of greenwashing. We have added a sentence to further clarify |
| 63. |  | p.19 line 63 | Given the challenges this is not unsurprising or unexpected. | We acknowledge that the challenges identified are not unexpected, as similar gaps have been observed in other sectors. However, we believe there is value in providing evidence through a robust review to advance the discussion on sanitation-specific adaptation strategies in Nairobi and Kenya. |
| 64. |  | p.19 line 80 | Useful description and references here that would fit more appropriately into an introduction. | This section summarises our analysis of the policy framework and we believe it is best placed in the results section |
| 65. |  | p. 20 line 81 | Unable to access the links (Reference to S1-S3) | OK |
| 66. |  | p.21 line 116 | What is this framework? | We give a detailed list of analysed documents in the tables in SI1-3 |
| 67. |  | p.23 line 169 | But this comes off a low base? 'Fantastic' is not an optimal description. | Agreed – we wanted to express that it is unlikely to be reached. Changed to fantastical |
| 68. |  | p. 26 line 257 | Overall, a useful commentary on what is known. The summary provides a better understanding against these 3 factors. | OK |
| 69. |  | p. 29 line 340 | This needs more attention both in the methods of extraction and analysis. | See our responses above |
