# Supplementary material for: Business-as-usual and fantasy planning – an analysis of equity within climate adaptation planning for sanitation in Nairobi
Source: PLoS One. 2025 Dec 30;20(12):e0339272. doi: 10.1371/journal.pone.0339272 (PMC12752985; doi:10.1371/journal.pone.0339272)
Supplement: Attachment — Submitted filename: Response to reviewer comments.docx [file pone.0339272.s007.docx]

**Reviewer #4:** The authors have modified the manuscript based on the previous suggestions but there are some minor adjustments to make this paper ready for publication. Here are some:

- **Comment 1:** Kindly ensure any framework mentioned is named and explicitly explained to convey the idea to potential reader. Explain why a particular framework is selected over the others.

**Response:** We understand the reviewer’s concern and have revised the text to explicitly name and explain the socio-technical regime framework. We have also clarified why this framework was selected, citing previous studies by Welie et al. (31) and Mdee et al. (30), who have already applied it to analyse Nairobi’s sanitation sector. Their work provides a strong conceptual foundation for our study (see lines 80 ff).

- **Comment 2:** While multiple data sources are used, on-ground validation appears limited

**Response:** We agree and explicitly acknowledge this limitation in the manuscript. Our analysis adopts a citywide approach, as literature on sanitation adaptation at this scale remains limited, particularly for cities with heterogeneous sanitation systems. Please also refer to our response to comment 4.
**Comment 3:** The transition is abrupt between the sections and technical terms need to be adequately explained.

**Response:** We suspect the reviewer might not have accessed figure 2 in which we define the sanitation regimes
**Comment 4:** Is there any on-ground validation of the satellite imagery/ shapefiles used in the research?

**Response:** Comprehensive on-ground validation was not feasible given the citywide scale of analysis. However, we validated our shapefiles by cross-referencing them with recent satellite imagery and an updated basemap (see lines 124 ff) In addition we consulted expert opinions. We also provide an example of local on-ground validation using for the Mukuru area (lines 255–259).

- **Comment 5:** Strengthen the justification for adapting Wood & Roelich (Table 2)

**Response:** We strengthened the justification given in lines 179ff to *As no coherent definition for ‘just’ or ‘equitable’ climate adaptation of urban sanitation currently exists, we drew upon environmental and energy justice scholarship (22). We adapted the energy justice framework proposed by Wood and Roelich (22) to the urban sanitation context by translating its justice dimensions into evaluative aspects of equitable sanitation adaptation (Table 2).*

- **Comment 6:** Detail the criteria used to assign a "dominant" regime to each area, especially where heterogeneity exists. How were overlaps or ambiguities resolved?

**esponse:** The process is described in Figure 1 We clearly state in the limitations that we acknowledge the impreciseness of our estimates. We have further sharpened this section (line 337ff): *We acknowledge that our estimates on sanitation service configuration across Nairobi have considerable limitations as they are derived from a necessarily simplified allocation of dominant sanitation service regimes to sub-areas. These classifications were informed by the prevailing technology and management arrangements identified within each area but inevitably obscure finer-grained overlaps, hybrid systems, and the coexistence of multiple service modalities. Consequently, both our spatial distribution and quantitative estimates should be viewed as indicative rather than definitive, acknowledging that each area reflects multiple and interacting service configurations shaped by broader political-economic processes (30).*

- **Comment 7:** Provide more specifics on "validation and refinement" using stakeholder input and studies. What discrepancies were found? How were they reconciled? A short appendix table summarizing validation sources and key adjustments would be valuable.

**Response:** We provide a description of our iterative approach to the sanitation regime classification in figure 1. We also provide a description of our steps to estimate the population in Annex 4 (SI). More detail is given in the text lines 122 – 145 as well as in the limitation section lines 341-347.

- **Comment 8:** Some few grammatical errors to be corrected include:
  - ***Comment 8a:*** Line 39: “It calls for an robust and honest…..” should be changed to “It calls for a robust and honest….”
  - **Response:** Changed as suggested
  - ***Comment 8a:*** Line 89-90: “on the sanitation sector of Nairobi and Kenya …” should be changed to “on the sanitation sector of Nairobi, Kenya.
  - **Response:** This is correct as it is written, we refer to both the studies specific to Nairobi and also more broader studies about the sanitation sector in Nairobi
